# Reinforcement learning of altruistic punishment differs between cultures and across the lifespan

**DOI:** 10.1371/journal.pcbi.1012274

**Published:** 2024-07-11

**Authors:** Ziyan Guo, Jialu Yu, Wenxin Wang, Patricia Lockwood, Zhen Wu

**Affiliations:** 1 Department of Psychological and Cognitive Sciences, Tsinghua University, Beijing, China; 2 Lab for Lifelong Learning, Tsinghua University, Beijing, China; 3 Centre for Human Brain Health and Institute for Mental Health, School of Psychology, University of Birmingham, Birmingham, United Kingdom; Dartmouth College, UNITED STATES OF AMERICA

## Abstract

Altruistic punishment is key to establishing cooperation and maintaining social order, yet its developmental trends across cultures remain unclear. Using computational reinforcement learning models, we provided the first evidence of how social feedback dynamically influences group-biased altruistic punishment across cultures and the lifespan. Study 1 (*n* = 371) found that Chinese participants exhibited higher learning rates than Americans when socially incentivized to punish unfair allocations. Additionally, Chinese adults showed slower learning and less exploration when punishing ingroups than outgroups, a pattern absent in American counterparts, potentially reflecting a tendency towards ingroup favoritism that may contribute to reinforcing collectivist values. Study 2 (*n* = 430, aged 12–52) further showed that such ingroup favoritism develops with age. Chinese participants’ learning rates for ingroup punishment decreased from adolescence into adulthood, while outgroup rates stayed constant, implying a process of cultural learning. Our findings highlight cultural and age-related variations in altruistic punishment learning, with implications for social reinforcement learning and culturally sensitive educational practices promoting fairness and altruism.

## Author summary

How do people from different cultures and ages learn to punish unfair behavior? Such punishment is crucial for societal cohesion, yet remains poorly understood. We tested participants from China and the USA, and from early adolescence to adulthood. Chinese individuals, compared to Americans, quickly associate punishing unfair actions with social feedback. However, they tend to hesitate when learning to punish ingroup members, unlike American individuals. Interestingly, this preference for ingroup members becomes more prominent as Chinese participants grow from adolescence into adulthood, reflecting a gradual adoption of collectivist values. These findings emphasize how cultural norms and developmental stages strongly influence the learning processes that shape social decision-making, offering insights for creating interventions that promote justice and cooperation in diverse societies.

## Introduction

Human cooperation, particularly the tendency to collaborate anonymously with unrelated individuals in large groups, presents a fascinating evolutionary conundrum. Central to understanding this phenomenon is altruistic punishment, wherein bystanders incur costs to punish cooperative norm violators without material gain. This mechanism is essential in promoting social harmony, fairness, and order [1–4]. Notably, altruistic punishment often exhibits parochialism amidst intergroup conflicts; adults typically mete out harsher punishments for outgroup violators, and are more likely to advocate for ingroup victims [5–8]. These biases underscore the profound impact of our group-centric nature on moral standards. However, empirical evidence is lacking in showing how these biases are learned and influenced by social norms across cultures and throughout the lifespan [9], although the norm-psychology account emphasizes the role of culturally transmitted cooperative norms and cognitive mechanisms [10,11]. Investigating the developmental trajectory of altruistic punishment can yield crucial insights into the nature of human morality, norms, and strategies to foster altruistic norm enforcement.

Social norms, as shared behavioral standards, regulate the expected conduct within a community [12–14]. The norm-psychology account posits that culture-specific norms, which individuals internalize and adhere to, influence parochialism in social behaviors [15]. For instance, in individualistic-oriented cultures, where egalitarian values are socially incentivized [2,16], people typically mete out equal punishments to unfair ingroup and outgroup members when making decisions with deliberate consideration [7,17]. In contrast, collectivist-oriented cultures, such as China, place greater importance on obligations to prioritize groups’ interests and maintain ingroup harmony [18–21]. Consequently, Chinese participants have been observed punishing ingroup transgressors less severely than outgroup offenders despite their negative attitudes toward unfair ingroups [9]. These findings highlight the significant influence of culturally specific norms on group bias in altruistic punishment. However, the learning processes underlying these behaviors, especially how individuals discern the appropriate response to norm violations during intergroup interactions remains an open question.

Previous research indicates that individuals learn social norms based on social feedback, including others’ suggestions, evaluations, and affects [22–24], and adjust their altruistic punishment accordingly [22,25]. Meanwhile, showing preferential treatment or favoring ingroup members often receives substantial positive social feedback, such as good reputations [26] and increased collaborations [27]. In individualistic cultures, principles of fairness and equality are often more highly valued and socially rewarded [28]. Consequently, anti-discrimination behaviors across groups are reinforced. In contrast, in collectivist cultures, individuals are often educated and socialized to prioritize the needs and interests of the group (e.g., family, school, society) over their personal interests [29]. For example, adolescents who prioritize the needs and interests of their group are highly appreciated by their parents, educators, and peers [30]. Such feedback serves as a potent reinforcement mechanism, encouraging individuals to consistently favor their own group, making it a deeply ingrained and socially reinforced behavior [31–33].

Notably, according to the norm-psychology account, once individuals have internalized their culturally ingrained norms, such norms become ends in themselves or part of individuals’ utility functions and motivate action regardless of other payoffs and sanctions [34], which can hinder the process of learning new social norms [31]. Research has shown that for those who have firmly internalized a social norm and developed specific behavioral tendencies, violating these norms can cause psychological discomfort, even when there are clear material benefits to doing so [35]. In addition, to maintain a positive self-image, individuals avoid situations that may cause them to deviate from their internalized social norms and established behavioral preferences [35]. Consequently, when individuals have established a stable ingroup bias for punitive behavior, external social feedback that encourages punishment of ingroup transgressors is less likely to be learned. This effect may be more pronounced in collectivist cultures that prioritize group harmony and group interests. However, although previous research has shed light on how internalized norms may block the adoption of new social norms [31,34,35], such studies have predominantly employed condition-comparison methods in behavioral tasks, which cannot capture the temporal dynamics of learning processes. Therefore, the current study primarily aims to examine how individuals from different cultural backgrounds dynamically learn to impose altruistic punishment in response to external social feedback and how their pre-existing biases affect the learning process.

To achieve this goal, we employed a computational approach using Reinforcement Learning (RL) models. Reinforcement learning theory illustrates how decisions are paired with outcomes over time, and elucidates how learning occurs via prediction error, the discrepancy between expected and actual outcomes. Prediction error is scaled by the learning rate, which indicates the extent to which an individual updates their beliefs or expectations based on new information or feedback [36,37]. By analyzing learning rates, we can delineate quantitative differences in how individuals learn to punish unfair ingroup and outgroup members, and illustrate how these learning mechanisms vary across cultures [38–41]. Furthermore, following recent studies [36,42] we can capture the pre-existing action bias (a bias to make a response that inhibits punishment of ingroup members, regardless of its expected outcome value), and examine how it impacts the learning process. Accordingly, we hypothesized that Chinese adults might exhibit a pronounced pre-existing ingroup action bias, reflecting their internalized cultural norms of ingroup preferences. Consequently, we anticipated that they might demonstrate lower learning rates when learning to punish ingroup (vs. outgroup) members. In contrast, influenced by individualistic values that emphasize fairness, Americans might exhibit a less pronounced pre-existing ingroup action bias. Therefore, we predicted that they would likely demonstrate similar learning rates for both ingroup and outgroup transgressions.

Another unaddressed question pertains to the developmental trajectory of altruistic punishment during intergroup interactions. Previous research suggests that in individualistic-oriented cultures, group bias in altruistic punishment may decrease with age [43,44], as children learn social norms against bias and discrimination [43,45,46]. For example, American children’s ingroup favoritism in altruistic punishment appears to decrease between the ages of 6 and 8 [44]. Additionally, older American children (age 9 and above) are more likely than younger children (age 6 and under) to rectify existing unequal resource allocations between socially disadvantaged outgroups (i.e., African Americans) and advantaged ingroups (i.e., European Americans), thereby promoting intergroup fairness [47]. This suggests that if social norms prioritize intergroup fairness over ingroup favoritism, group bias in altruistic punishment may diminish with age due to the socialization process. This hypothesis warrants further investigation, especially since there is currently a reliance primarily on WEIRD (Western, Educated, Industrialized, Rich, Democratic) samples [43,45,46]. The developmental trajectory of altruistic punishment may differ in collectivist-oriented cultures such as China, where ingroup loyalty is more internalized and socially incentivized than egalitarian values. Moreover, as individuals age, they are likely to become more integrated into their cultures [48], absorbing and reinforcing the norms and values that emphasize ingroup harmony and cohesion [29]. Consequently, we hypothesize that for Chinese participants, the pre-existing ingroup bias for punishing ingroup members may intensify with age, resulting in a decreased learning rate for punishing ingroup members. This phenomenon may reflect an increased reluctance to act against one’s ingroup due to the greater internalization of collective values over time.

Furthermore, while research has highlighted cultural differences in the adoption of altruistic punishment, research gaps remain regarding the role of individual differences in explaining these learning differences. Previous work has shown that the process by which individuals learn and enforce social norms can be significantly influenced by the extent to which they perceive themselves as belonging to a particular social group, a concept referred to as group identity salience [49,50]. Empirical research indicates that individuals in collectivist cultures, which prioritize group interests, are more likely to identify with their group than those in individualistic cultures [51]. Furthermore, individuals with strong group identification are more likely to act in accordance with internalized group norms [52] and are reluctant to adopt social norms that conflict with their internalized norms [53–55]. In contrast, individuals who are willing to abandon norms are often less identified with their groups [56]. Therefore, we hypothesize that individual differences in group identity may explain the observed cultural differences in learning altruistic punishment. Specifically, compared to American participants, Chinese participants are more likely to form a strong group identity as a result of experimental manipulation involving group membership, and consequently exhibit a culturally ingrained in-group bias in punishment. Such a pre-existing bias may further reduce the learning rates of social feedback among Chinese participants relative to their American counterparts.

**Fig 1:**
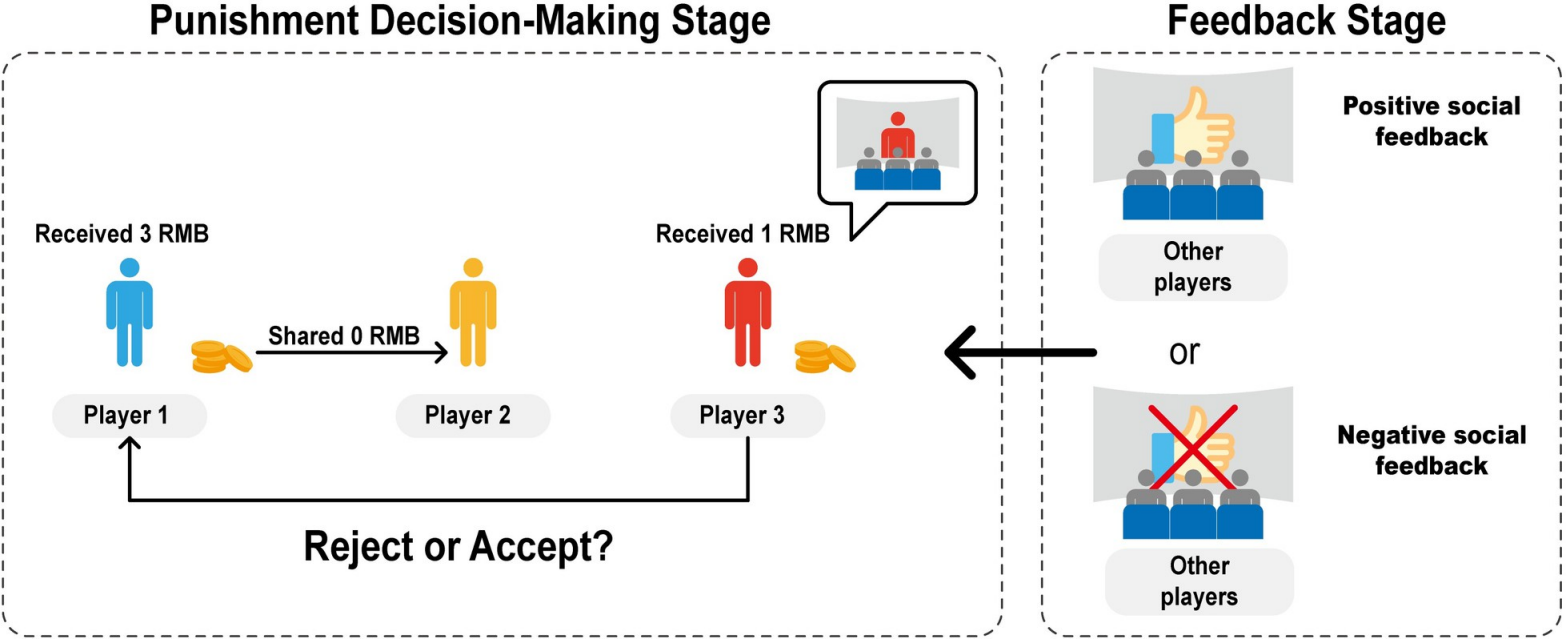
Reinforcement Learning Altruistic Punishment Task. Participants selected a team color (yellow) and were assigned as Player 3, with Players 1, 2, and observers computer-generated. As shown in the left panel, during the punishment decision-making stage, Player 1 chose to share 0 RMB with Player 2. Endowed with 1 RMB, Player 3 then chose to either accept or reject the allocation, with rejection equating to a costly punishment. As shown in the right panel, in the feedback stage, Player 3 received social feedback from the observers in the waiting room, conveyed by a thumbs-up for positive feedback or a thumbs-up overlaid with a red cross for negative feedback. The feedback was based on a 60% approval threshold. Participants were assigned to either the ‘punishment-encouragement’ or ‘acceptance-encouragement’ condition, incentivizing either punishment or acceptance with an 80% chance of positive feedback and a 20% chance of negative feedback, respectively.

To examine how learning to impose altruistic punishment varies across cultures and throughout the lifespan, we devised a novel reinforcement learning altruistic punishment paradigm (Fig 1). In this framework, participants (acting as third parties) could either accept observed allocations or punish unfair players at a cost. The unfair players may be members of the participant’s own ingroup or outgroup. This distinction is created through the minimal group paradigm, which prompts participants to choose to join a team based on their color preference [50,57]. Such minimal groups are devoid of the emotional bonds, learning opportunities, and historical contexts that characterize real-world social groups, thus helping to control for extraneous variables. Despite their simplicity, research has demonstrated that these groups effectively reveal individuals’ general ingroup bias, which predicts ingroup favoritism in various real-life groups [57,58]. Subsequently, participants received trial-by-trial feedback on other observers’ evaluations of their punitive decisions. In the "punishment-encouraged" condition, punishing had an 80% likelihood of receiving positive feedback (thumbs up) from other players, while accepting only had a 20% likelihood. In the "acceptance-encouraged" condition, these associations were reversed. This design allowed us to observe how individuals dynamically adjusted their behavior based on social feedback, with a particular focus on responses to unfair allocations committed by ingroup versus outgroup members. Crucially, these contingencies were not explicitly instructed but were implicitly learned through trial and error, enabling participants to update their decisions based on feedback from other observers. Using computational modeling, we analyzed the dynamic adjustments people made to altruistic punishment during intergroup interactions based on external social feedback. This novel approach stands apart from previous reinforcement learning paradigms in which participants learn arbitrary associations between actions and outcomes, such as associating a picture with rewards. In contrast, the associations in our study have real-life social significance, such as encouraging the punishment of ingroup selfishness or not, which taps into individuals’ internal values regarding group identity and fairness. By integrating the behavioral task with computational modeling, we developed a tool to probe into the dynamic reinforcement learning mechanisms that underpin norm enforcement across different cultures and age groups—an area that remains relatively uncharted.

Addressing this knowledge gap, we conducted two studies. In Study 1, we compared reinforcement learning parameters between Chinese and American participants, quantifying how manipulated social feedback interacts with cultural norms to shape individuals’ altruistic punishment behaviors. Study 2 further investigated the developmental trajectory of group bias in altruistic punishment among Chinese participants, spanning from adolescence to adulthood, especially considering the limited data on non-WEIRD samples. We hypothesized that: (1) learning to punish ingroup and outgroup members could be computationally differentiated; (2) during the learning process, both the pre-existing action bias and learning rates would show more pronounced group distinction among Chinese participants than among American participants; (3) ingroup bias in altruistic punishment among Chinese participants would increase with age, while the learning rate for punishing ingroups would decrease with age; and (4) The observed cultural differences in learning altruistic punishment may be explained by individual differences in group identity.

## Results

The study recruited 389 adults (217 Chinese, 172 American) online and 213 adolescents offline (see Methods; total *N =* 602). Study 1 investigated whether the learning mechanisms underlying ingroup bias in punishment behavior differed across cultures. We analyzed participants’ altruistic punishment towards ingroup and outgroup members among the Chinese and American participants, using computational reinforcement learning models to estimate learning rates (α) and temperature (β) parameters, as well as the constant bias term. Study 2 further examined the development of ingroup bias across different age groups among Chinese participants.

### Chinese participants show greater ingroup bias in altruistic punishment than American participants

**Fig 2:**
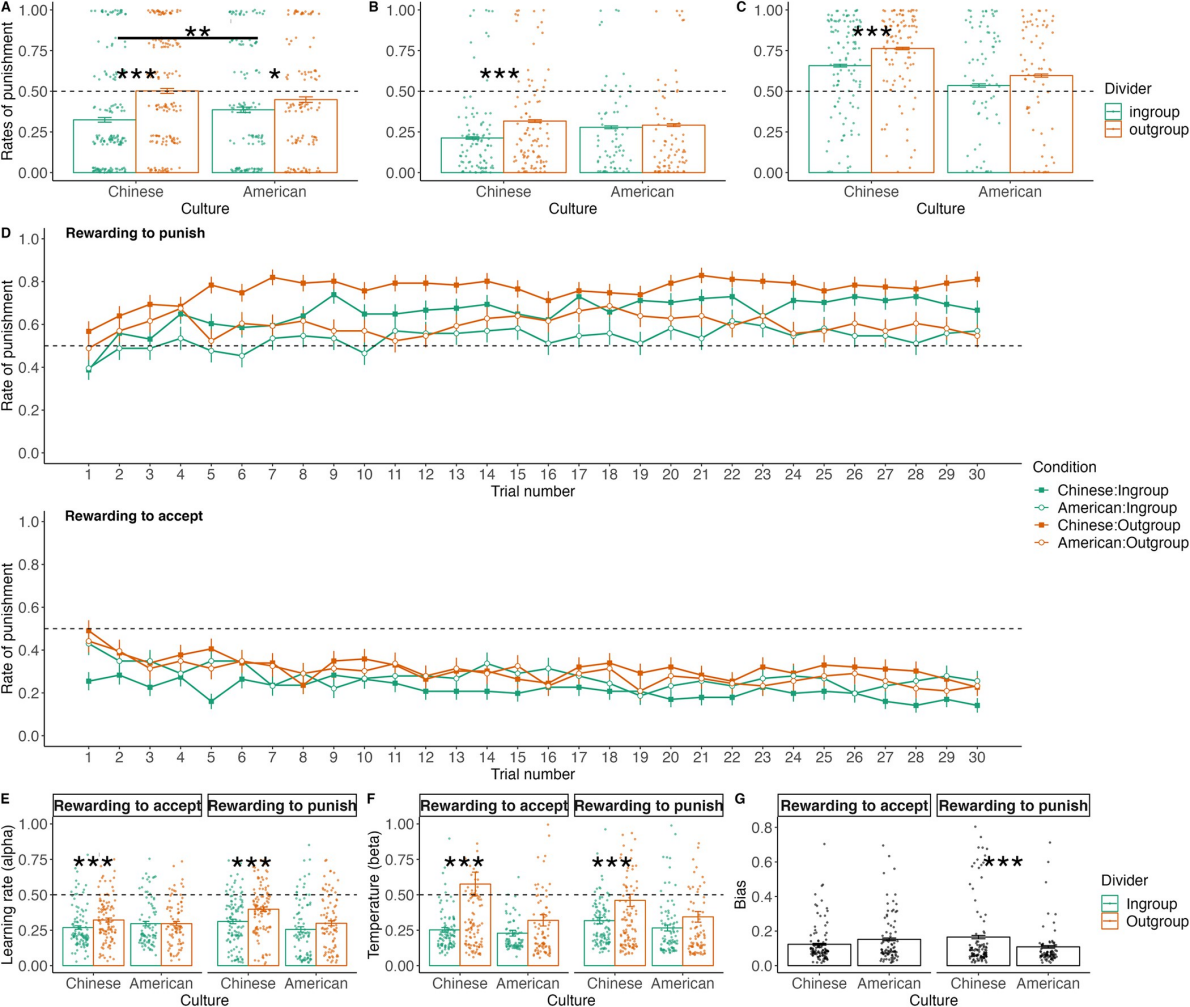
Cultural and group influences on altruistic punishment behavior in pre-test and reinforcement learning stages. **A** Pre-test stage: Chinese participant exhibited stronger ingroup bias compared to American participants. Overall, participants punished ingroup (green) more severely compared to outgroup (orange) dividers. However, Chinese participants exhibited an enhanced group bias (*b* = - 0.82, *SE* = 0.29, *z* = -2.94, *p* = .003). **B** Reinforcement learning stage: when acceptance was rewarded, Chinese participants punished ingroup dividers (green) less severely compared to outgroup dividers (*b* = -0.91, *SE* = 0.23, *z* = -4.06, *p* < .001), no significant differences were found between punishment towards ingroup and outgroup dividers among American participants (*b* = -0.20, *SE* = 0.26, *z* = -0.76, *p* = .871). **C** Reinforcement learning stage: when punishment was rewarded, Chinese participants punished ingroup dividers (green) less severely compared to outgroup dividers (*b* = -0.87, *SE* = 0.22, *z* = -4.01, *p* < .001), no significant differences were found between punishment towards ingroup and outgroup dividers among American participants (*b* = -0.61, *SE* = 0.26, *z* = -2.37, *p* = .084). **D** Group level trial-by-trial punishment rates in the two divider conditions (ingroup: green, outgroup: orange) for each culture group (Chinese: squares, American: circles). Points represent group mean; error bars are standard errors. **E** Regardless of whether punishment or acceptance was rewarded, Chinese participants had lower learning rates for ingroup norms (*b* = -0.07, *SE* = 0.01, *t* = -5.51, *p* < .001), while American participants showed no differences (*b* = -0.02, *SE* = 0.01, *t* = -1.46, *p* = .460). **F** Regardless of whether punishment or acceptance was rewarded, Chinese participants had lower β when learning ingroup norms (*b* = -0.23, *SE* = 0.04, *t* = -5.85, *p* < .001), while no differences were observed for American participants (*b* = -0.08, *SE* = 0.04, *t* = -1.90, *p* = .231). **G** Chinese participants showed a stronger bias compared to American participants, especially when punishment was rewarded (*M*_Diff_ = 0.06, *SE* = .01, *t* = 5.11, *p <* .001). Error bars represent standard errors. * *p* < .05, *** *p* < .001.

First, we assessed the influence of culture and the divider’s group membership on participants’ punishment decisions (0 = accept, 1 = punish) during the pre-test stage using a GLMM model. Fixed effects were culture and the divider’s group membership, and by-participant random intercepts, as well as the random slope for divider’s membership, were also included to further reduce the probability of committing false-positive errors (see Method for the detailed model selection procedure and S1 Table for the model selection process). Additionally, to control for potential confounding demographic differences between American and Chinese samples, we included age, gender, education level, and subjective socioeconomic status as covariates in the analyses. Results revealed a significant interaction between culture and divider groups (Fig 2A; see S2 Table for the complete model results). Pairwise comparisons showed that both Chinese and American participants punished ingroup members less severely compared to outgroup members (Chinese: *b* = -1.44, *SE* = 0.19, *z* = -7.69, *p* < .001; American: *b* = -0.61, *SE* = 0.22, *z* = -2.80, *p* = .026). Notably, Chinese participants exhibited a more pronounced ingroup bias compared to their American counterparts (*b* = - 0.82, *SE* = 0.29, *z* = -2.94, *p* = .003). This finding aligns with previous research [7] and indicates the role of cultural values in shaping group biases in altruistic punishment. In addition, we assessed participants’ beliefs about the game’s authenticity to rule out the possibility that differences in these beliefs might contribute to different levels of ingroup bias across groups. Analysis revealed no significant differences in beliefs about the game’s authenticity among culture groups (*F* (2, 599) = 0.27, *p* = .765, η_p_^2^ = 0.001; Chinese adults: *M =* 3.91, *SD* = 1.70; American adults: *M =* 3.87, *SD* = 1.61; Chinese adolescents in study 2: *M =* 3.78, *SD* = 1.98).

Next, we investigated whether participants adjusted punitive behavior in response to social feedback during the reinforcement learning stage, and whether this learning was contingent on trial-by-trial feedback. Average punishment rates were computed across trials, as a function of culture (Chinese vs. American), divider group membership (ingroups vs. outgroups), and social feedback (punishment-encouragement vs. acceptance-encouragement) (Fig 2B and 2C). Participants from both cultures displayed higher punishment rates than chance level (50%; all *t*s > 2.28, *p*s < .05) when rewarded to punish and lower punishment rates than chance level when rewarded to accept (50%; all *t*s < -8.28, *p*s < .001). Further analysis of the trial-by-trial punishment rates showed a positive association with the trial number when punishment was rewarded (*b* = 0.04, *SE* = 0.01, *t* = 3.11, *p* = .003) and a negative association when acceptance was rewarded (*b* = -0.03, *SE* = 0.01, *t* = -3.90, *p* < .001) (Fig 2D). Taken together, these results suggest that individuals can assimilate punishment norms through social feedback.

Then we examined potential differences in punishment behavior across cultures, divider groups, and punishment norms during the reinforcement learning stage (see S3 Table for the model selection process and S4 Table for the complete model results). A GLMM analysis uncovered a significant interaction between cultures and divider groups (*b* = -0.49, *SE* = 0.24, *z* = -2.03, *p* = .042) (Fig 2B and 2C). Pairwise comparisons showed that Chinese participants punished ingroup dividers less severely compared to outgroup dividers, regardless of whether punishment (*b* = -0.87, *SE* = 0.22, *z* = -4.01, *p* < .001) or acceptance was rewarded (*b* = -0.91, *SE* = 0.23, *z* = -4.06, *p* < .001). In contrast, no significant differences were found between punishment towards ingroup and outgroup dividers among American participants, whether punishment was rewarded (*b* = -0.61, *SE* = 0.26, *z* = -2.37, *p* = .084) or acceptance was rewarded (*b* = -0.20, *SE* = 0.26, *z* = -0.76, *p* = .871). Consistent with the pre-test stage, the ingroup bias in punishment behavior was more pronounced among Chinese participants during the reinforcement learning stage. These results highlight that despite being exposed to a similar new environment and receiving identical social feedback, individuals from different cultural backgrounds may interpret or respond differently due to their ingrained cultural norms, which persistently influence their learning processes.

Nevertheless, solely focusing on mean punishment behavior does not elucidate how ingroup bias unfolds throughout the learning process. On the one hand, regarding the social feedback learning process, the observed fewer punishment behaviors towards ingroups could arise from distinct interpretations of external feedback. This could manifest as slower updating of values to punish ingroups (indicated by lower learning rates α). Alternatively, despite learning values from the environment, participants may follow the learned values less closely (indicated by higher temperature β). On the other hand, beyond the social feedback learning process, individuals may demonstrate a stable action bias that leads to leniency towards ingroups, showing a resistance to learning social feedback. To dissect these possibilities, we conducted computational modeling analyses to inspect the underlying mechanisms.

### Learning to punish ingroup and outgroup selfishness differs computationally

**Fig 3:**
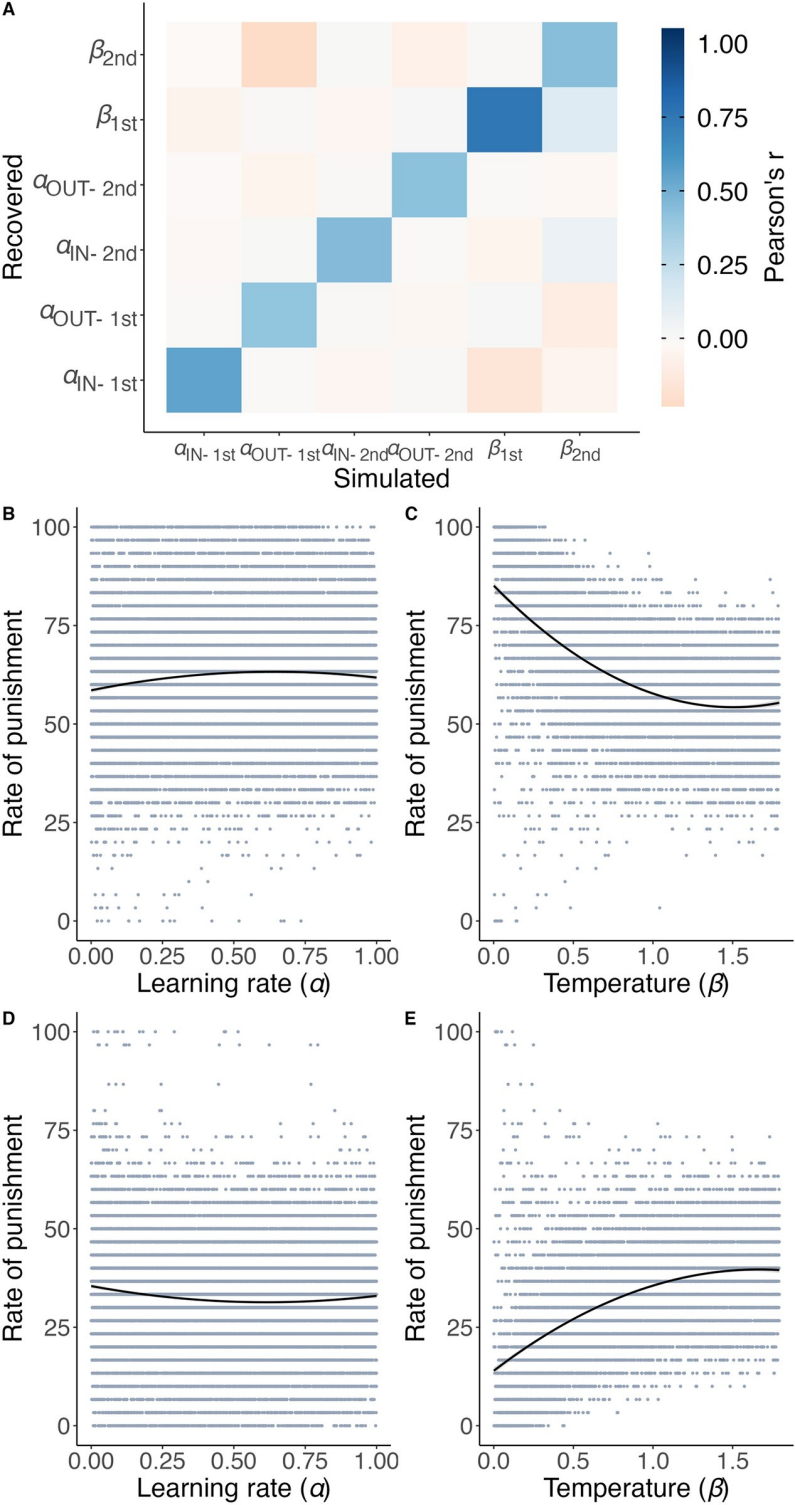
Computational modeling reveals distinct norm learning and decision-making processes in punishing ingroups and outgroups. **A** Parameter recovery analysis for the winning 4α2β + bias model demonstrates the recoverability of the parameters. Data from 15,625 simulated participants were used, with a confusion matrix depicting correlations between simulated and fitted parameters. Enhanced colors represent higher values. See S6 Table for the source data. **B** When punishment was rewarded, a simulation experiment illustrated the quadratic relationship between α and the rate of punishment. An α of 0.64 was identified as the point at which the rate of punishment was maximized. **C** Quadratic relationship between β and the rate of punishment. A β of 1.51 was identified as the point at which the rate of punishment was minimized. **D** When acceptance was rewarded, a simulation experiment illustrated the quadratic relationship between α and the rate of punishment. An α of 0.61 was identified as the point at which the rate of punishment was minimized. **E** Quadratic relationship between β and punishment rates. A β of 1.66 was identified as the point at which the rate of punishment was maximized.

We fitted computational models of reinforcement learning to estimate the parameters of learning rates (α) and temperature (β), as well as the constant bias term (see Methods). The learning rate (α) captured how individuals learned and modified their value expectations concerning punishments based on external feedback. Temperature (β) reflected how individuals adjusted their behavior during decision-making based on these value expectations. Furthermore, to depict individuals’ deeply ingrained cultural preferences, which were resistant to changes in punishment based on social feedback, we integrated a persistent bias term into our model. This bias term was designed to capture the default bias of individuals that showed resistance to social feedback. Then we employed the Integrated Bayesian Information Criterion (Integrated BIC) to compare seven candidate models (see Methods). In samples combining both Chinese and American participants, as well as in separate analyses, a model with distinct α for ingroup and outgroup dividers across two blocks, separate β for dividers, and a bias term (4α2β + bias model) provided the best fit for participants’ choices (see S5 Table), suggesting a computationally distinct learning process for punishing ingroups versus outgroups. To further validate our computational model, we conducted a parameter recovery (see Methods) using the winning 4α2β + bias model, thereby demonstrating the recoverability of the parameters (see Fig 3A and S5 Table).

Moreover, although a higher learning rate typically suggests that learning is driven mainly by recent feedback, and a higher temperature implies more randomness across trials, emerging evidence increasingly suggests that the relationship between these parameters and behavioral choices is heavily dependent on the experimental design [59–61]. This suggests that the extent to which higher learning rates led to increased punishment in contexts where social feedback encouraged such responses or, conversely, to decreased punishment in contexts that encouraged acceptance, remained an open question. Therefore, building on previous research, we utilized simulated experiments and compared empirical data from our experiments to enable a clear interpretation of the relationship between parameters (learning rate and temperature) and punishment behaviors across different experimental conditions. By simulating data from 5000 participants using the 4α2β model with a bias term under both conditions of punishment-encouragement and acceptance-encouragement, we produced 10,000 simulated participants and 40,000 α values, fully encompassing the potential range from 0 to 1. We calculated the punishment rates as the percentage of punishment enforced by the same participant, averaged across blocks for each divider group.

Our findings revealed that simulated α values exerted quadratic impacts on punishment rates in conditions that encouraged punishment. Specifically, in the punishment-encouragement condition, the simulation experiment demonstrated a trend where punishment rates first increased and then decreased as a function of the learning rate (α), with an optimal learning rate of 0.64 maximizing punishment rates (see Fig 3B). However, when further examining the relationship between learning rates and rates of punishment in the empirical data, we found the average α values among Chinese (*M* = 0.28, *SD* = 0.24) and American (*M* = 0.21, *SD* = 0.24) adults in study 1 and Chinese adolescents in study 2 (*M* = 0.41, *SD* = 0.23) were below the optimal learning rate of 0.64. In fact, 85.3% of participants’ α values in study 1 and 79.4% of participants’ α values in study 2 were found to be below 0.64. These results demonstrated that in our empirical study, a higher learning rate represented faster adaptation to increased punishment in response to external feedback encouraging such behavior. These results were further supported by the positive correlation between higher α values and punishment rates in empirical data for adults in study 1 (*r* = 0.29, *p* < .001) and adolescents in study 2 (*r* = 0.26, *p* < .001).

Conversely, in conditions that encouraged acceptance, the simulated experiment revealed an initially declining and then increasing trend in punishment rates with increasing α, indicating a learning rate of 0.61 that minimized punishment rates (see Fig 3D). Meanwhile, analysis of empirical data elucidated that the mean α values for both Chinese (*M* = 0.30, *SD* = 0.19) and American (*M* = 0.30, *SD* = 0.21) adults in study 1, and Chinese adolescents in study 2 (*M* = 0.42, *SD* = 0.23), were below the learning rate of 0.61. Notably, 88.7% of participants’ α values in study 1 and 81.0% of participants’ α values in study 2 were below this threshold. Moreover, the higher α values were negatively related to punishment rates under conditions encouraging acceptance (study 1: *r* = -0.21, *p* < .001; study 2: *r* = -0.14, *p* < .001), suggesting that a higher learning rate might reflect quicker adjustment to reduce punishment according to external feedback.

Next, when examining the relationship between temperature and punishment rates, we found quadratic effects of temperature on punishment rates (see Fig 3C). Under conditions that encouraged punishment, simulations showed punishment rates decreasing and then increasing with rising β, indicating a β of 1.51 for minimizing punishment. Empirical data revealed mean β values for both Chinese (*M* = 0.39, *SD* = 0.37) and American (*M* = 0.31, *SD* = 0.28) adults in study 1, and Chinese adolescents in study 2 (*M* = 0.42, *SD* = 0.23) below the 1.51 threshold, with 98.2% of the β values in study 1 and 93.8% of the β values in study 2 under this threshold. Temperature was negatively correlated with punishment rates (study 1: *r* = -0.08, *p* < .001; study 2: *r* = -0.27, *p* < .001), suggesting higher temperatures decrease the likelihood of actions aligning with social feedback that encourages punishment.

Finally, under conditions that encouraged acceptance, the simulation showed punishment rates rising then falling with temperature (β), peaking at a β of 1.66 (see Fig 3E). Empirical findings indicated average β values for Chinese *(M* = 0.30, *SD* = 0.19) and American (*M* = 0.30, *SD* = 0.21) adults in study 1, and Chinese adolescents in study 2 (*M* = 0.42, *SD* = 0.23) below the 1.66 threshold, with 97.4% of the β values in study 1 and 93.2% of the β values in study 2 under this threshold. There was a significant positive relationship between temperature and punishment rates (study 1: *r* = 0.23, *p* < .001; study 2: *r* = 0.46, *p* < .001), suggesting that higher temperatures reduced the likelihood of decisions consistent with social feedback that encourages acceptance.

Taken together, these results revealed that in our research, higher learning rates represented accelerated social feedback learning, allowing quicker adaptation in punishment or acceptance as encouraged. Higher temperatures were correlated with increased behavioral randomness, implying a rise in decision-making variability.

### Chinese participants show lower learning rates for punishing ingroups than American participants

We examined the impact of culture, divider groups, and punishment norms on learning rates (α) and temperature (β) in reinforcement learning. Regarding α, faster learning occurred in the first block (*M* = 0.36, *SD* = 0.24) than in the second (*M* = 0.26, *SD* = 0.19; *t* = -8.75, p < .001). Linear mixed model (LMM) analyses revealed a significant two-way interaction between divider group membership and cultural background across two blocks (*b* = -0.09, *SE* = 0.04, *t* = -2.58, *p* = .010) (Fig 2E; see S7 Table for the model selection process and S8 Table for the complete model results). Chinese participants had lower learning rates for ingroup norms (*b* = -0.07, *SE* = 0.01, *t* = -5.51, *p* < .001), while American participants showed no significant differences (*b* = -0.02, *SE* = 0.01, *t* = -1.46, *p* = .460).

Regarding β, we also found a significant two-way interaction between divider group membership and cultural background (*b* = - 0.14, *SE* = 0.06, *t* = -2.48, *p* = .014) (Fig 2F; see S9 Table for the model selection process and S10 Table for the complete model results). Chinese participants had lower β for ingroup norms (*b* = -0.23, *SE* = 0.04, *t* = -5.85, *p* < .001), while no differences were observed for American participants (*b* = -0.08, *SE* = 0.04, *t* = -1.90, *p* = .231). These results uncover distinct learning mechanisms underpinning punitive behavior between Chinese and American participants, despite both showing an ingroup bias in their behavioral choices. When learning how to punish ingroup (vs. outgroup) members, Chinese participants adjusted their punishment slower in response to external feedback, as evidenced by their lower learning rates. They also demonstrated a less random decision-making process with a tendency to consistently select options with the highest expected value for ingroup members, reflected in their lower β values. This value-driven strategy in decision-making involving ingroup members among Chinese adults contributes to the observed ingroup bias in punishment behavior.

Furthermore, we examined the impact of culture and punishment norms on the bias term, which shifts individuals’ preference towards accepting ingroup members’ unfair allocations and thus captures individuals’ default bias that exhibits resistance to social feedback. We found a significant interaction between culture and punishment norms (*F*(1, 385) = 7.25, *p* = .007, η_p_^2^ = 0.018; see Fig 2G). Under the punishment-encouragement condition, Chinese participants showed a stronger bias compared to American participants (*M*_Diff_ = 0.06, *SE* = .01, *t* = 5.11, *p <* .001), whereas the bias was not significantly different across cultures under the acceptance-encouragement condition (*M*_Diff_ = -0.03, *SE* = .01, *t* = -2.55, *p =* .053). These results indicated that Chinese (vs. American) participants exhibited more resistance to social feedback when they were rewarded for punishing ingroups.

Taken together, our data suggested that, compared to their American counterparts, Chinese adults demonstrated a consistent preference in treating ingroup members and were less inclined to adjust their decisions in response to dynamic external feedback about ingroup members. These findings revealed the potential influence of ingrained cultural norms on the blocking of learning from social feedback. Given that theoretical and empirical research indicates cultural norms are gradually internalized and acquired throughout development, it is plausible that such ingroup bias in learning is nurtured alongside individual development within a specific culture. Specifically, if Chinese participants increasingly internalize collectivist cultural norms emphasizing ingroup preference, this results in a more deeply rooted ingroup bias. Consequently, their responsiveness to external feedback regarding the punishment of ingroup members decreases with age, leading to a decrease in learning rates for such punitive measures. On the contrary, due to a lack of cultural norms on how to treat outgroup members, we expect no significant developmental changes in adjusting punitive behaviors based on feedback, manifesting in stable learning rates for punishing outgroup members across different ages.

### Chinese participants’ ingroup bias in punishment increases with age from adolescence to adulthood

**Fig 4:**
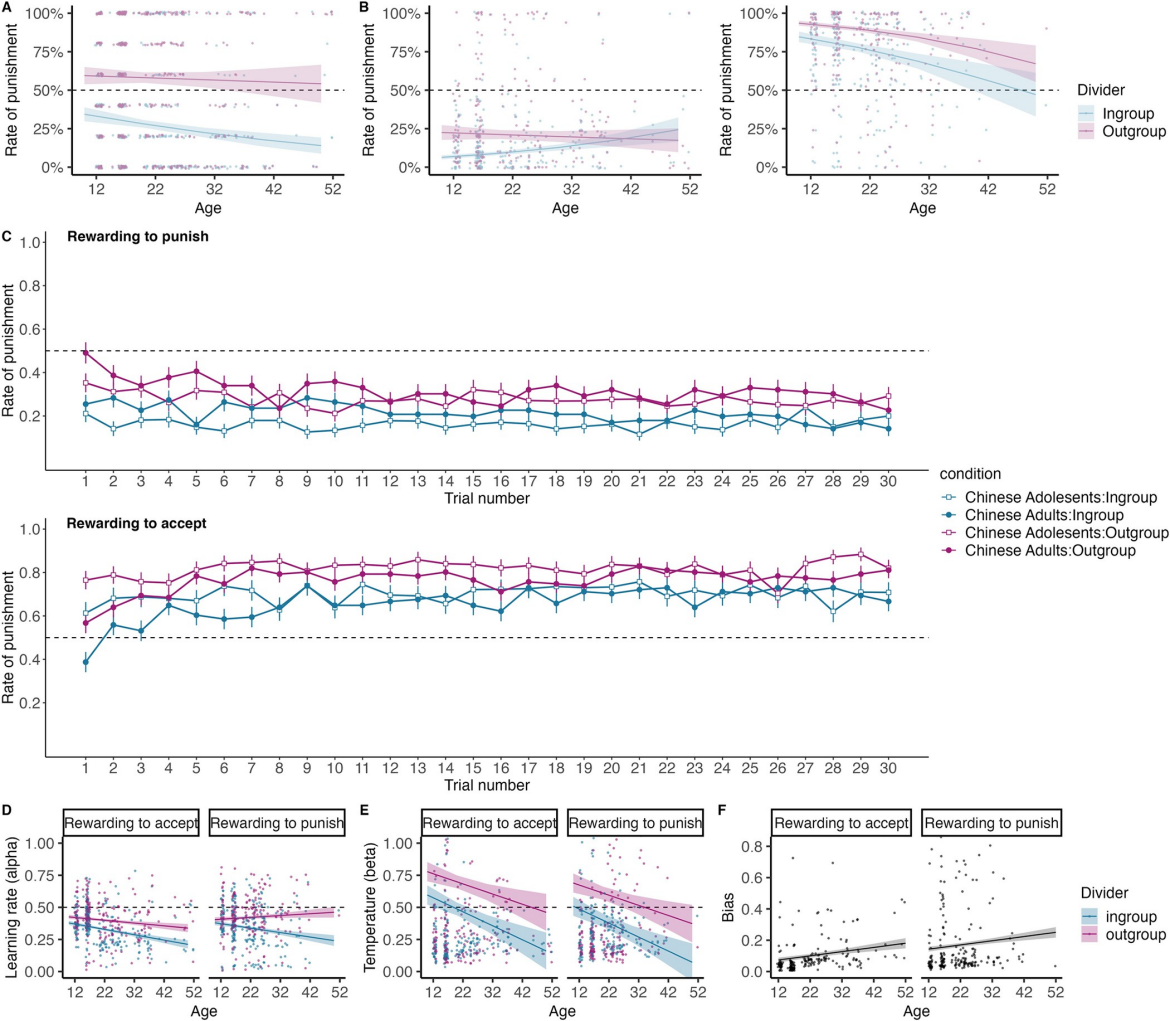
Age-related differences in ingroup bias and learning mechanisms among Chinese individuals. **A** Pre-test stage: the interaction effect between age and dividers’ group reveals a decrease in punishing unfair ingroup dividers (blue) with increasing age (*b* = -0.03, *SE* = 0.02, *z* = -2.05, *p* = .040), while punishment rates for outgroup dividers (purple) remain unchanged (*b* = -0.01, *SE* = 0.02, *z* = -0.34, *p* = .734). **B** Reinforcement learning stage: younger participants’ behavioral responses toward ingroups were more consistent with the norms than were older participants: when acceptance was rewarded, younger participants exhibited less punishment compared to older individuals (*b* = 0.03, *SE* = 0.02, *z* = 2.32, *p* = .020); when punishment was rewarded, younger participants exhibited more punishment than older participants *b* = -0.03, *SE* = 0.02, *z* = -1.51, *p* = .131). By contrast, the age differences were not significant for unfair outgroup dividers under either social feedback condition. **C** Trial-by-trial punishment rates for ingroup (blue) and outgroup (purple) dividers across age groups (adolescents: squares, adults: circles). Points represent group mean, and error bars are standard errors. **D** learning rate (α) for punishing unfair ingroups decreased with age (*b* = -0.14, *SE* = 0.03, *t* = -4.21, *p* < .001), while the α for punishing outgroups was not associated with age (*b* = -0.02, *SE* = 0.03, *t* = -0.68 *p* = .500). **E** Temperature (beta): Lower β for learning ingroup norms than outgroup norms regardless of age (*b* = -0.25, *SE* = 0.05, *t* = -5.36, *p <* .001); β decreased with age (*b* = -0.12, *SE* = 0.04, *t* = -2.74, *p =* .006). **F** the bias term significantly increased with age regardless of punishment norms (*b* = 0.13, *SE* = 0.05, *t* = 2.65, *p* = .008). Error bars represent standard errors. * *p* < .05. ** *p* < .01. *** *p* < .001.

Study 2 examined age-related differences in the reinforcement learning processes of punitive decisions in Chinese individuals aged 12–52. First, we examined the influence of the dividers’ group and participants’ age on punitive decisions (0 = accept, 1 = punish) during the pre-test stage. A GLMM model revealed a significant interaction between age and the group of dividers (*b* = -0.03, *SE* = 0.01, *z* = -2.62, *p* = .009) (Fig 4A; see S11 Table for the model selection process and S12 Table for the complete model results). To unpack the two-way interaction effect, pairwise comparisons indicated a decrease in punishing unfair ingroup dividers as age increased (*b* = -0.03, *SE* = 0.02, *z* = -2.05, *p* = .040). However, age was not significantly related to punishment rates for unfair outgroup dividers (*b* = -0.01, *SE* = 0.02, *z* = -0.34, *p* = .734). These results suggest that the Chinese participants’ group bias in altruistic punishment increases with age, and this increasing bias is primarily driven by ingroup favoritism rather than outgroup harshness. This pattern suggests that collectivist cultural values emphasizing ingroup favoritism become more embedded as individuals age.

We then tested age differences in punitive behavior during the reinforcement learning stage. Using a GLMM analysis, we identified a significant interaction between age and the group of dividers (*b* = 0.03, *SE* = 0.01, *z* = 2.05, *p* = .041) (Fig 4B and 4C; see S13 Table for the model selection process and S14 Table for the complete model results). Younger participants punished ingroups less than their older counterparts when acceptance was rewarded (*b* = 0.03, *SE* = 0.02, *z* = 2.32, *p* = .020), but tended to punish more when punishment was rewarded (*b* = -0.03, *SE* = 0.02, *z* = -1.51, *p* = .131). This implies that younger Chinese individuals were quicker to adapt to environmental changes in response to ingroup transgressions, and such adaptation tended to decline with age. By contrast, outgroup punishment remained stable in both conditions (punishment-encouragement: *b* = -0.17, *SE* = 0.13, *z* = -1.41, *p* = .158; acceptance-encouragement: *b* = -0.02, *SE* = 0.10, *z* = -0.25, *p* = .800). Thus, our study provides the first evidence that the prior observed ingroup bias in the literature [26,51,62] is mainly driven by increasing tolerance for ingroup norm violations with age, rather than intensifying harshness towards outgroups.

### Learning rates decrease with age for punishing ingroups, but preserve for punishing outgroups

Next, consistent with Study 1, in samples combining both Chinese adults and adolescents, as well as in separate analyses, a model with distinct α for ingroup and outgroup dividers across two blocks, separate β for dividers and a bias term (4α2β + bias model) provided the best fit for participants’ choices (see S15 Table), indicating a distinct computational process for punishing ingroups and outgroups. Upon comparing the learning rates across two blocks, the first block demonstrated a significantly higher learning rate (*t* = 17.34, *p* < .001), denoting faster learning. As in study 1, we then used LMM to analyze the effects of age, group membership of dividers, and punishment norms on learning rate. LMMs revealed a significant two-way interaction between the group membership of dividers and age across two blocks (*b* = -0.06, *SE* = 0.02, *t* = -2.99, *p* = .003) (Fig 4D; see S16 Table for the model selection process and S17 Table for the complete model results). Crucially, the learning rate for ingroup punishment norms decreased with age (*b* = -0.14, *SE* = 0.03, *t* = -4.21, *p* < .001), while the α for outgroup punishment norms remained stable (*b* = -0.02, *SE* = 0.03, *t* = -0.68, *p* = .500). These results indicate that participants learned how to punish unfair ingroups more slowly compared to outgroups, and this difference became more prominent in older participants, implying a slower adaptation in punitive behavior towards ingroup members among older participants.

Additionally, when further looking into the adolescent group (aged 12–18), results revealed adolescents’ heightened sensitivity to social feedback during norm learning (see S18 Table for the model selection process and S19 Table for the complete model results). The adolescent group (aged 12–18) exhibited higher learning rates when socially incentivized to accept unfairness (*b* = 0.50, *SE* = 0.11, *t* = 4.66, *p* < .001) compared to adults (aged above 18). This enhanced learning rate was consistent across the 12–18 age group (*b* = 0.01, *SE* = 0.01, *t* = 0.64, *p* = .522). Notably, when comparing ingroup and outgroup transgressions, there was no significant difference in these learning rates for the adolescent group (*b* = 0.02, *SE* = 0.02, *z* = 1.01, *p* = .312), a contrast to adult participants. Considering that adolescence is characterized by pronounced neurodevelopment and concerns about social evaluation, this age difference may imply a sensitive window for social norm learning during adolescence.

We then investigated the influences of age, group membership, and punishment norms on temperature (β). An LMM analysis revealed only the main effect of age (*b* = -0.12, *SE* = 0.04, *t* = -2.74, *p =* .006) and dividers’ group membership (*b* = -0.25, *SE* = 0.05, *t* = -5.36, *p <* .001) was significant, showing that Chinese participants’ temperature was lower when learning norms regarding ingroups, and the temperature decreased with age (Fig 4E; see S20 Table for the model selection process and S21 Table for the complete model results). This finding suggests that in a collectivist culture, a value-driven behavioral adjustment towards ingroup members was well-established by adolescence.

Furthermore, we examined the impact of age and punishment norms on the bias term. We found the bias significantly increased with age regardless of punishment norms (*b* = 0.13, *SE* = 0.05, *t* = 2.65, *p =* .008; see Fig 4F). These results suggested a cultural inclination towards leniency for in-group members that becomes more pronounced over time.

Taken together, Study 2 provides initial evidence suggesting the role of collectivist culture in shaping ingroup bias in punitive decisions and learning mechanisms among Chinese individuals. Prior studies have indicated that group bias in third-party punishment may be driven by both a preference for ingroup members and an exclusion of outgroup members [8,51,62]. However, it remains unclear whether, in collectivist cultures as opposed to individualistic ones, the greater group bias in third-party punishment is driven more by one aspect over the other. Our findings indicate that with increasing age, Chinese participants exhibit an enhancement of pre-existing ingroup bias, accompanied by a reduction in the learning rate for punishing ingroup members. This observation suggests an evolving internalization of collectivist cultural norms that favor ingroup preference as individuals age. Conversely, the learning rate for punishing outgroup members does not follow a similar age-related pattern. These findings may suggest that a cultural bias against outgroups is not as strongly emphasized within collectivist cultures, potentially enabling a more adaptable punitive approach towards outgroups. These insights advance our understanding of how cultural values shape social decision-making and learning processes across various developmental stages.

### Group identity modulates learning from altruistic punishment

**Fig 5:**
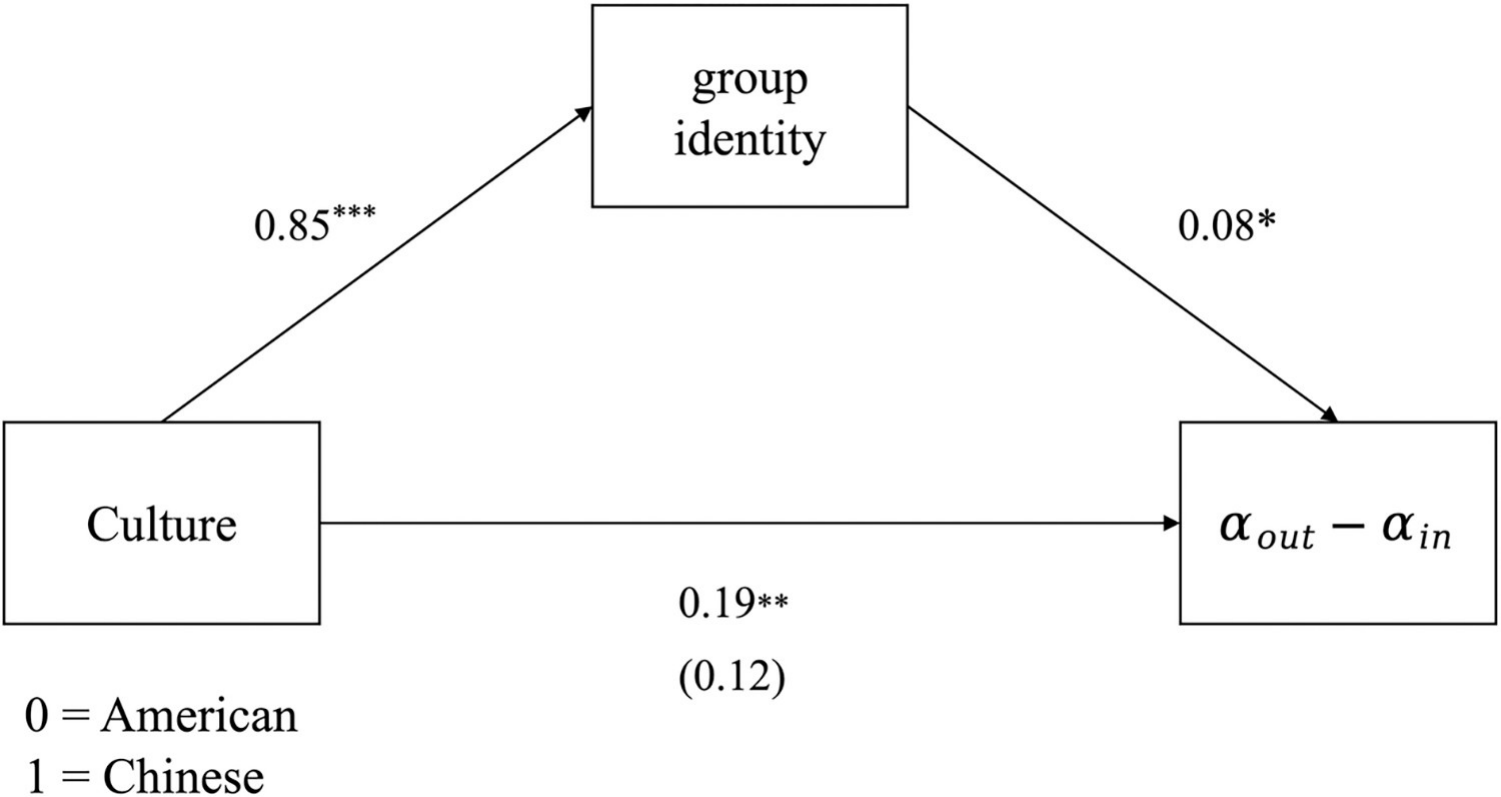
Indirect effect of culture on group bias in learning (α-out minus α-in) via group identity. Participants’ self-reported group identity mediated the relationship between culture and group bias in learning rates (standardized indirect effect = 0.07, 95% CI [0.003, 0.140], *p* = .039).

Subsequently, we tested whether individual differences in self-reported group identity could explain cultural differences of ingroup bias in learning rates. A standard mediation model was implemented using the mediation package in R. As depicted in Fig 5, our findings revealed that group identity significantly predicted ingroup bias in learning rate (α-out minus α-in, *b* = 0.08, *SE* = 0.04, *t* = 2.05, *p* = .041). Furthermore, a significant indirect effect of group identity (standardized indirect effect = 0.07, 95% CI [0.003, 0.140], *p* = .039, simulation = 5,000) indicated that group identity mediated the relationship between culture and group bias in learning rates.

## Discussion

Altruistic punishment is a crucial altruistic behavior that promotes cooperation. While people frequently demonstrate an ingroup bias, punishing outgroups more than ingroups when enforcing cooperative norms, how individuals dynamically adjust these biases in response to social feedback remains largely underinvestigated. In this study, we integrate behavioral tasks and reinforcement learning computational models to investigate the influence of social feedback on group-biased altruistic punishment across cultures and age groups. Our method offers a nuanced, quantitative analysis of cultural differences in learning how to punish ingroup and outgroup norm violators, and deepens our understanding of the development of group-biased altruistic punishment.

Our investigation revealed that Chinese individuals strongly adhere to ingroup loyalty norms when engaging in altruistic punishment. Analyzing pre-test punishment data without external social feedback revealed that Chinese participants showed a more significant ingroup bias in their punishment decisions compared to Western counterparts. This ingroup bias in punitive decisions among Chinese participants intensified with age, signifying a deepening process of social norm internalization over the course of individual development. These findings resonate with extant literature emphasizing the profound influence of social norms on punitive actions [22,63,64]. Within collectivist societies, individuals often receive positive social feedback, such as praise or reputation boosts, for fulfilling social roles that maintain group harmony [28]. This feedback serves as a potent reinforcement mechanism, encouraging internalized ingroup norms. As shown in prior research [7], Chinese adults reported guilt for ingroup members’ violations but still exhibited less severe punitive actions upon reflection. In contrast, the cultural norms in individualistic societies like the United States, which encourage a more personal approach to morality and behavior, could lessen the impact of ingroup loyalty on altruistic punishment.

More importantly, in addition to replicating the documented ingroup bias in altruistic punitive behaviors in a non-WEIRD sample, we provided the first evidence that punishing ingroups and outgroups involves distinct *learning* mechanisms across cultures. Specifically, Chinese participants exhibited a greater pre-existing action bias that inhibits them from punishing ingroup violators, and a group-biased learning process with lower learning rates and temperature for punishing ingroups than outgroups. In contrast, such group differentiation was not evident among American adults. These findings suggest that compared to American individuals, Chinese individuals adhered to a relatively steadfast set of ingroup loyalty norms in altruistic punishment, and such an ingrained norm impacted the learning of external social feedback regarding how to enforce punishment.

This aligns with the cultural evolutionary theory, which emphasizes the significant role of norm internalization in shaping human behavior [12,31,34,65]. The theory posits that natural selection has shaped humans to internalize norms, as such internalization decreases the costs related to information collection, processing, and decision-making, which are essential for ensuring cooperation [31,34]. However, this process of internalization also reduces behavioral flexibility, making it challenging for individuals to alter deeply ingrained norms [12,34,66]. For example, individuals frequently overestimate the difficulty of transitioning to a novel norm, with shifts occurring smoothly only in environments that provide effective feedback [35]. Additionally, to maintain a positive self-view, people typically avoid situations that might lead them away from their deeply held social norms and behaviors [35]. Although prior studies have highlighted how internalized norms might hinder adopting new norms, little is known about the temporally dynamic nature of learning processes. In contrast, our reinforcement model, which contains separate learning rates for different groups and a persistent bias term, quantified how individuals from different cultural backgrounds dynamically learn to impose altruistic punishment in response to external social feedback. It also identified how their pre-existing biases impact the learning process. Moreover, our study further supports cultural evolutionary theory by demonstrating that participants in collectivist cultures are reluctant to alter their behavior or adopt new norms when faced with social feedback that challenges the established way of treating ingroup members. It is plausible that such reluctance acts as a cultural filter, allowing only practices that align with existing values to persist and ensuring cultural stability [11,67].

Our analysis further revealed that self-reported group identity mediated this cultural difference, offering an individual-level perspective on cross-cultural variations in learning mechanisms. This finding is consistent with prior research, which indicates that the social norm learning process is markedly influenced by group identity. Specifically, Chinese participants, compared to Americans, showed a greater inclination to form group identities even within arbitrary minimal groups [7,68]. Moreover, those with a stronger identity were less inclined to adopt external social feedback regarding the punishment of ingroup (vs. outgroup) members, with heightened pre-existing ingroup bias and slower learning rates on ingroup (vs. outgroup) members. This is consistent with studies indicating that individuals with a strong identification with their groups are more likely to internalize norms [53–55] and may resist learning norms perceived as conflicting with their group’ s interests [54,55]. In conclusion, our study contributes to the empirical evidence of ingroup bias by revealing a computational mechanism driving group-biased norm enforcement in different cultures. This offers valuable insights for understanding and enforcing social norms cross-culturally.

Furthermore, we observed an increased ingroup bias among Chinese individuals as they aged. This was characterized by a decrease in the learning rates for punishing ingroups, which spanned from adolescence to older adulthood. Conversely, the learning rates for punishing outgroups remained stable. This finding contrasts with previous results obtained from Western cultures that suggest a reduced ingroup favoritism with age [44,45]. Our finding suggests that the collectivist culture, compared to the individualistic culture, fosters relatively more consistent internal values for ingroup norms across the lifespan. Consistent with cultural evolution theory [48,66], this trajectory suggests that individuals increasingly adopt their culture’s established norms and values with age. In the Chinese context—characterized by collectivism [68–71]—this trend indicates that older individuals, after prolonged exposure to social feedback, internalize norms favoring ingroup members more deeply. This likely reflects a cumulative social learning trajectory, where norms, reinforced through repeated interactions and feedback, may become stabilized with age [72,73]. Consequently, older adults are less likely to adjust punitive behaviors toward ingroup members in response to temporary social feedback. In contrast, the stable learning rates for punishing outgroups across age groups suggest that ingroup bias is driven more by growing favoritism toward ingroups with age rather than increasing hostility toward outgroups. This helps to clarify the theoretical debate regarding the formation of group bias [50].

Our findings also highlight adolescents’ heightened sensitivity to social feedback. Given that adolescence is characterized by pronounced neurodevelopment and concerns about social evaluation [74,75], it suggests that this may be a sensitive period for social norm learning. In collectivist cultures like China, which prioritize group harmony, this sensitivity could underpin the internalization of norms that favor ingroup members. Recognizing this sensitivity underscores the importance of tailored educational strategies. Previous research has demonstrated the effectiveness of bystander interventions in reducing bias-based bullying during adolescence [43,76]. However, adolescents often exhibit restrained prosocial bystander behaviors, and their willingness to intervene against ingroup members’ unfair behavior diminishes with age [77–79]. Thus, fostering a social environment that promotes altruistic punishment is crucial [80,81]. Schools can leverage social incentives to cultivate such environments.

A salient feature of our study centers on how individuals’ pre-existing social experiences shape reinforcement learning within specific social contexts. In contrast to prior reinforcement learning studies employing probabilistic tasks, where actions arbitrarily corresponded with outcomes (e.g., selecting an image might lead to a reward) [82–84], our paradigm incorporates a socially contextualized learning process where actions bear moral and social significance. This methodology has greater ecological validity, providing insights into the intricate social-cultural learning dynamics. Moreover, it holds potential in shedding light on dysfunctional reward processing, which is central to the pathophysiology of mental disorders like depression [84] and anxiety [85]. Such mental disorders frequently result in socially maladaptive behaviors, including impairments in understanding, following, and enforcing social norms [86]. A probable contributor to these issues is a deficiency in social reward learning. Despite this, the bulk of existing research on reward learning in mental disorders has been confined to cognitive dimensions employing monetary incentives [87]. To achieve a comprehensive understanding of the factors influencing adaptive social decision-making, it is critical to focus on the role of reward learning during social interactions.

The limitations of this study and its findings suggest avenues for further research. First, our study highlights the significant differences in learning altruistic punishment across cultures and ages, even in arbitrarily assigned groups. Research indicates that the ingroup bias observed in minimal groups can predict biases across diverse real-world social groups, including families, friendships, and ethnic communities [57,58]. Nevertheless, the significant variability in social decision-making among these groups underscores the need for further investigation into how social feedback learning influences decision-making within natural groups [57,58]. Second, following common practice in cross-cultural research, our study employs country as a proxy for culture, which may not fully account for confounding variables between the Western and Chinese samples [71]. Despite demographic similarities in our samples, unaddressed socio-ecological factors (e.g., community size, pathogen prevalence) may still influence ingroup bias and norm learning [2,15]. Future research would benefit from accounting for these elements to better disentangle the cultural effects. Third, our findings suggest the influence of cultural and developmental factors on altruistic behavior. However, the causality between these elements remains unclear. It is recommended that future research should manipulate cultural norms and employ longitudinal methods in order to establish causality and trace the development of these phenomena. Fourth, our study did not include fair trials in order to streamline the learning process. However, future studies should investigate how participants switch their learning of punishment between mixed contexts (e.g., fair vs. unfair conditions) through more complex learning strategies, such as model-based learning [88,89].

In sum, our unique approach allowed us to illuminate the potential causal effect of social feedback on group-biased altruistic punishment, and elucidate the distinct computational learning mechanisms across cultures and age groups. This approach facilitated an exploration of the interplay between experimentally manipulated norms and culturally ingrained norms, which are typically persistent and have deep roots. Our study particularly reveals distinct reinforcement learning processes across different cultures: Chinese adults were overall more responsive to social feedback than their American counterparts, and adjusted their decisions more slowly when punishing ingroup members than outgroups. Notably, this pattern was absent among Americans, indicating a pronounced ingroup bias in collectivist cultures. Furthermore, this ingroup bias is primarily driven by an increasing ingroup favoritism from adolescence to adulthood, rather than outgroup harshness, contributing to the debate over the formation of group bias. Our findings highlight the theoretical significance of how socio-cultural norms impact the underlying learning mechanisms during the social decision-making processes, and provide valuable insights for future studies to assess practical implications and effectiveness in fostering altruistic norm enforcement behaviors in a culturally sensitive manner.

## Materials and methods

### Ethics statement

Experiments in this study were approved by the Research Ethics Committee, Department of Psychology, Tsinghua University, China (ref: 2021–26). For adult participants, as all tasks were completed online, participants provided their consent by ticking checkboxes after reading the information sheet and prior to the administration of tasks on computers. For child participants, formal written consent was obtained from both the children and their guardians.

### Participants

For the power analysis, we utilized the G*Power software package (version 3.1.9.7). In Study 1, consistent with prior research [7,17], we assumed a small-to-medium effect size (Cohen’s *f* = .14, α = .05, power [1 –β] = 95%) to detect the interaction effect of the group membership of dividers and culture, which resulted in a total sample size of 405 participants. Therefore, we initially recruited 440 participants; however, we excluded 51 participants who did not complete the main altruistic punishment task, resulting in a final sample of 389 participants (see Table 1 for the demographic information). Specifically, Chinese participants (*n* = 217, *M*age = 28.01, *SD*age = 7.46; 69 males) were recruited online through Credamo [7], while Western participants (*n* = 172, *M*age = 35.01, *SD*age = 9.90; 89 males) were recruited online through Prolific [90]. Informed consent was obtained from all participants before the commencement of the study. In study 2, to examine how ingroup preferences among Chinese participants develop with age, we recruited 213 adolescent participants (*M*age = 15.01, *SD*age = 1.82, range from 12 to 18; 108 males) and combined their data with the data from 217 adult Chinese participants in Study 1, resulting in a total of 430 participants. The study method was the same as in Study 1.

**Table 1:** Demographic information of Chinese and American adults in Study 1 and Chinese adolescents Study 2.

	Chinese adults	American adults	Chinese adolescents
Age in years, *M* (*SD*)	28.01 (7.46)	35.01 (9.90)	15.98 (1.82)
Gender, *n* (% Male)	69 (32.8%)	89 (51.7%)	108 (50.7%)
Subjective socioeconomic status, *M* (*SD*)	5.18 (2.43)	5.76 (1.93)	5.73 (1.72)
Education level, *M* (*SD*)	1.98 (0.90)	2.54 (0.97)	1(0)

### Altruistic punishment reinforcement learning task

The present study aimed to investigate how individuals dynamically adjust their behavior in response to external feedback, specifically in the context of altruistic punishment. To this end, we employed a commonly used altruistic punishment task and used computational modeling of reinforcement learning to capture participants’ trial-by-trial behavior changes. The task consisted of two stages: (1) Punishment Decision-Making and (2) Feedback.

*Punishment Decision-Making*. In this stage, the game involved three players. Players 1 and 2 each contributed equally and earned a reward of 3 RMB by correctly solving three calculation problems. Player 1 was then given the reward and had the option to allocate half of it (1.5 RMB) to Player 2 or keep it all. Player 3, endowed with 1 RMB, observed Player 1’s allocation and could choose to accept or reject it. A rejection by Player 3 would result in both Player 1 and Player 3 receiving 0 RMB, categorizing this action as costly altruistic punishment. If Player 3 accepted the allocation, both Player 1 and Player 3 retained their respective endowments. Additionally, Player 3’s choices had no impact on Player 2’s endowments; that is, even if Player 3 chose to punish Player 1’s unfair allocation, Player 2 did not receive any compensation and only received 0 RMB. This approach aimed to directly assess the effect of punishment, without the influence of compensation motives.

*Feedback*. Immediate social feedback was provided to Player 3 regarding their decision to punish or accept the allocation. Player 3 was informed that their decisions would be observed by other players in the waiting room, who would provide feedback during each round. Positive social feedback (a thumbs-up) was given when more than 60% of the other players approved of Player 3’s choice, while negative feedback (a thumbs-up with a red cross) was given when less than 60% of the other players approved. Participants were randomly assigned to one of the two social norm conditions: the reward-to-punish or reward-to-accept condition. In the reward-to-punish condition, participants were incentivized to punish: punishment resulted in positive feedback with an 80% probability and negative feedback with a 20% probability. In the reward-to-accept condition, participants were incentivized to accept unfair allocations: acceptance resulted in positive feedback with an 80% probability and negative feedback with a 20% probability.

### Manipulation of group membership

To examine group bias in third-party punishment (TPP), we manipulated the divider’s group membership (ingroup vs. outgroup) using the minimal group paradigm [50,57]. Participants chose either a blue, yellow, or red team based on color preference. This strategy is widely utilized in social psychology research and aids in minimizing the influence of participants’ varying previous experiences with actual social groups [50,57]. Furthermore, aligning with prior research [7,17], we ensured Player 2 belonged to a different team from Player 1 and Player 3, making the receiver an outgroup member from the viewpoint of participants.

### Procedure

First, participants chose to join a team. Then they were informed that they would be randomly assigned to be Player 1, 2, or 3 and would interact with other players anonymously, and their decisions in the game would affect their payoffs. Indeed, all participants were assigned to be Player 3, and other players, including Players 1 and 2, and observers in the waiting room, were all simulated by programming. Player 1 could be either an ingroup or outgroup member of Player 3. Player 2 was always from a different team than Player 1 and Player 3.

Next, the experiment started and consisted of three phases: pre-test, reinforcement learning, and post-test. In the pre-test phase, participants made decisions without receiving feedback, and were told that players in the waiting rooms did not know their choices. There were 10 trials in the pre-test phase, all of which were unfair allocations, with five trials for each ingroup and outgroup condition. These decisions served as a baseline for altruistic punishment.

Next, participants completed the reinforcement learning phase, in which they received probabilistic feedback after each decision. There were two blocks for each ingroup and outgroup condition, with 15 trials per block, resulting in a total of 60 trials. The order of ingroup and outgroup conditions was randomized across participants.

Finally, in the post-test phase, participants completed ten trials without feedback, including five trials for each ingroup and outgroup condition, to determine whether the effect of the feedback lasted. Besides, we exclusively used unfair allocation scenarios across all trials, for the following two reasons. First, since fair allocations are generally accepted with little variability in responses, they offer limited scopes for learning through social feedback. By focusing on unfair distributions, we aimed to enhance the scope for learning. Second, including fair allocations could require participants to adapt to varying conditions, thereby complicating the learning process with more advanced strategies like model-based reinforcement learning. Therefore, by concentrating solely on unfair scenarios, our experiment avoided these complexities, facilitating a clearer analysis of the responses to unfairness. After completing the game, to provide an index of group identity, participants rated the extent to which they identified to their group on a 7-point scale (“*How much do you feel that you belong to this group*?”, 1 = very low, 7 = very high). Besides, to assess the experiment’s authenticity, participants evaluated their belief in the real-time interaction with other players during the game (“*How much do you believe that you interacted with other players online in real time*?”, 1 = very low, 7 = very high). Then participants reported their demographic information including gender, age, education level (1 = not a high school graduate, 5 = graduate school/postgraduate training) and subjective socioeconomic status (SES). The SES was measured using the social ladder task from 1 (bottom) to 10 (top) [71]. A higher score on the scale indicates that participants perceive their economic status to be more favorable relative to others in society.

### Computational modeling

Computational modeling was conducted using MATLAB. As in previous studies, we modeled the data using an R-W reinforcement learning model.


$$V_{t+1}(a)=V_{t}(a)+\alpha_{i(j)}∙PE_{t}$$ (1)

The model estimated how each participant updated the expected values (V) of each choice (punish or accept) based on the trial-by-trial feedback they had received (see Eqs 1 and 2). The V values were initialized at each participant’s probability of choosing each option in the pre-test phase, ranging from 0 to 1. The subscript i in the equation represented the divider group (ingroup vs. outgroup), while subscript j represented the order of blocks (1^st^ vs. 2^nd^). The learning rates were scaling parameters that adjusted the amplitude of value changes from one trial to the next.


$$PE_{t}=R_{t}-V_{t}(a)$$ (2)

After each trial, the value of the chosen option was updated based on the prediction errors (PE, see Eq 2) of the chosen option. The PEs were computed as the difference between the current reward received (R = 1 if positive feedback, R = 0 if negative feedback) and the expected value of the chosen option, thus PEs represent the extent to which environmental rewards diverge from expected outcomes.

The softmax link function was utilized to model the relationship between the expected value of a particular action *V*_*t*(*a*)_, and the probability of choosing that action on trial t:

$$P_{t}(a|V_{t}(a))=\frac{e^{{(V}_{t}(a)/\beta )}}{\sum_{a^{\prime }}e^{{(V}_{t}(a\prime )/\beta )}}$$ (3)

Crucially, the decision calculus is refined by the ingroup bias term *Bias*_(*i*,*a*)_ as introduced into computations through the softmax function which determines the selection probability for each action.


$$P_{i,t}(a|V_{t}(a))=\frac{1}{1+\sum_{a^{\prime }\neq a}e^{-(V(a)-V_{t}(a^{\prime })+Bias_{\left(i,a\right)})/\beta }}$$ (4)

The bias term, *Bias*_{(*i*, *a*)}, is dynamically applied and is contingent on the divider group subscript *i* and the action subscript *a* (see Eq 5).


$$Bias_{\left(i,a\right)}=\{\begin{array}{cc} bias & \mathrm{if}\;i=\mathrm{ingroup}\;\mathrm{and}\;a=\mathrm{accept}\\ 0 & \mathrm{otherwise} \end{array}$$ (5)

Specifically, for the ingroup condition, the bias term is added to the value deviations between the choice of acceptance and punishment, thereby increasing the likelihood of opting for acceptance while simultaneously decreasing the propensity to select punishment. This means that for ingroup members, irrespective of whether the norm is to punish or to accept, the bias specifically applies to increase the tendency to accept ingroup members’ behaviors, indicating a potential inclination to follow the ingrained cultural preference. For the outgroup condition, the bias term does not influence the deviation between values for various choices, reflecting an absence of particular cultural bias toward outgroup members.

β represents the temperature. A high β value indicates that choices appear random, with equal likelihood of choosing each option regardless of expected value, while a low β value results in consistently selecting the option with the highest expected value across all trials.

### Model fitting and comparison

Models were fitted using a hierarchical approach and compared using Integrated Bayesian Information Criterion (Intergated BIC). We tested multiple models that varied with respect to whether learning could be explained by shared or separate free parameters across group members (ingroup and outgroup dividers) and block numbers (first and second block). We examined whether shared or separate learning rates and temperature, in particular, resulted in a better model fit. Specifically, we compared six candidate models:

- 1α1β: one α and one β for all group dividers and blocks;
- 2α1β: one α for ingroup dividers and one α for outgroup dividers; one β for all group dividers and blocks;
- 2α2β: separate α and β for both ingroup and outgroup dividers;
- 4α1β: separate α for both ingroup and outgroup dividers in the first and second blocks; one β for both ingroup and outgroup dividers;
- 4α2β: separate α for both ingroup and outgroup dividers in the first and second blocks; one β for ingroup dividers and one β for outgroup dividers;
- 4α2β + bias: separate α and β for both ingroup and outgroup dividers in the first and second blocks, with a bias term

To examine the association between parameters (learning rate and temperature) and punishment rates for our task, the present study utilized a simulation approach to obtain data from a large sample of 10,000 participants. Specifically, we drew the learning rates (α) and temperature (β) from beta and gamma distributions, respectively. Simulated participants had a distinct learning rate for each divider group (ingroup vs. outgroup) and block (first vs. second), ranging from 0 to 1 to produce 40,000 learning rate values. Moreover, consistent with previous research [83], we evaluated our winning model’s reliability via parameter recovery on simulated data. Simulating choices 15,625 times using our experimental schedule and employing an iterative maximum a posteriori (MAP) approach fitting revealed strong correlations (see Fig 3A) between simulated and fitted parameter values, indicating effective model parameter estimation in our experiment.

The MAP approach offers improvements over traditional maximum likelihood estimation (MLE) through a hierarchical analysis comprising individual subject assessments and collective sample evaluations. Initially, we set uninformative priors with means μ = 0.1 (plus noise) and a variance σ^2^ = 100 the group-level Gaussian distributions. First, during the expectation step, we calculated the log-likelihood of the anticipation’s series of choice given a model M and its parameter vector η_i_(α and β) for each participant i (i∈[1, N]). We summed the conditional probability of each trial’s choice given the model’s parameter η_i_ and initial expectation of values for both punishment and non-punishment $V_{i}^{\prime }=[V_{a};V_{b}]$ over all trials. The prior probability of each participant’s η_i_ was calculated given the group-level Gaussian distributions over the parameters (with mean of μ and variance of σ^2^). Given the probability of the already observed choice series is constant, we computed the posteiror probability (P_*posterior*,*i*_) estimate as follows:

$$P_{posterior,i}=\underset{\eta_{i}}{\max}\left[\sum_{t}\left(\log(p_{t}(choice_{t}|\eta_{i},V_{i}^{\prime }))+\log\left(normpdf(\eta_{i}|\mu ,\sigma^{2})\right)\right)\right]$$

$$\eta_{i}=argmax_{\eta_{i}}\left[\sum_{t}\left(\log(p_{t}(choice_{t}|\eta_{i},V_{i}^{\prime }))+\log\left(normpdf(\eta_{i}|\mu ,\sigma^{2})\right)\right)\right]$$

Second, during the maximization step, we recomputed μ and σ^2 based on the estimated set of η and their Hessian matrix H (as calculated with Matlab’s fminunc) over all N participants.

$$\mu =\frac{1}{N}\sum_{i}\eta_{i}$$

$$\sigma^{2}=\frac{1}{N}\sum_{i}[\eta_{i}^{2}+diag(pinv(H_{i}))]-\mu^{2}$$

Where the diagonal terms of the inverted Hessian matrix (computed in Matlab with diag(pinv(H_i_))) give the second moment around η_i_, approximating the variance, and thus the inverse of the uncertainty with which the parameter can be estimated. We repeated expectation and maximization step until convergence—defined as a posterior likelihood change under 0.001 between iterations—or a maximum of 800 iterations were completed. Free parameters that were bounded underwent careful transformation using link functions, such as sigmoid transformations for learning rate parameters, to maintain estimate precision.

### Statistical analysis

We used R Version 4.1.0 (80) to conduct all statistical analyses on behavioral data and computational modeling parameters. Models were all run using the package ‘lme4’. We employed generalized linear mixed models (GLMM) to analyze the decisions in altruistic punishment game due to binary responses (punish = 1; accept = 0), and we used linear mixed-effects models (LMM) to analyze the continuous learning rates (α). In Study 1, fixed terms included cultural background, dividers’ membership and punishment norms. Age, gender, education level, and subjective socioeconomic status were included as covariates to control potential confounding demographic differences between American and Chinese samples. In Study 2, fixed terms included age, dividers’ membership and punishment norm. Gender, education level, and subjective socioeconomic status were included as covariates. The model selection process began with the full model, which incorporated random intercepts for each participant and random slopes for all fixed within-group factors [91]. We then fitted a series of reduced models, each excluding one of the random slopes, and selected the model with the lowest Akaike Information Criterion (AIC). This model was subsequently compared to the more complex original using a likelihood-ratio test. We opted for the more complex model if the p-value from the χ^2^-statistic was less than 0.2 [92]. If not, we continued to simplify the model until an optimal one was chosen or all random slopes were removed. Models that failed to converge were excluded from this analysis. For two-level factors, we applied sum contrasts—for example, for group membership (ingroups = -0.5, outgroups = 0.5), cultural background (Chinese = -0.5, American = 0.5), norms regarding punishment (punishment-encouragement = -0.5, acceptance-encouragement = 0.5), order of blocks (first block = -0.5, second block = 0.5), and gender (male = -0.5, female = 0.5). Additionally, we centered all continuous predictors, such as age, education level, and subjective socioeconomic status, around their mean values. Data and analysis scripts can be obtained online (https://doi.org/10.5281/zenodo.11239516).

## Supporting information

S1 Table Model comparison and the model selection process for punishment behaviors in pre-test stage in Study 1. (DOC)

S2 Table Model results for punishment behaviors in pre-test stage in Study 1. (DOC)

S3 Table Model comparison and the model selection process for punishment behaviors in learning stage in Study 1. (DOC)

S4 Table Model results for punishment behaviors in learning stage in Study 1. (DOC)

S5 Table Study 1 Model Comparison Results. (DOC)

S6 Table Parameter Recovery Results. (DOC)

S7 Table Model comparison and the model selection process for learning rates in Study 1. (DOC)

S8 Table Model results for learning rates in Study 1. (DOC)

S9 Table Model comparison and the model selection process for temperature in Study 1. (DOC)

S10 Table Model results for temperature in Study 1. (DOC)

S11 Table Model comparison and the model selection process for punishment behaviors in pre-test stage in Study 2. (DOC)

S12 Table Model results for punishment behaviors in pre-test stage in Study 2. (DOC)

S13 Table Model comparison and the model selection process for punishment behaviors in learning stage in Study 2. (DOC)

S14 Table Model results for punishment behaviors in learning stage in Study 2. (DOC)

S15 Table Study 2 Model Comparison Results. (DOC)

S16 Table Model comparison and the model selection process for learning rates in Study 2. (DOC)

S17 Table Model results for learning rates in Study 2. (DOC)

S18 Table Model comparison and the model selection process for adolescents’ learning rates in Study 2. (DOC)

S19 Table Model results for adolescents’ learning rates in Study 2. (DOC)

S20 Table Model comparison and the model selection process for temperature in Study 2. (DOC)

S21 Table Model results for temperature in Study 2. (DOC)
