# Supplementary material for: Reinforcement learning of altruistic punishment differs between cultures and across the lifespan
Source: PLoS Comput Biol. 2024 Jul 11;20(7):e1012274. doi: 10.1371/journal.pcbi.1012274 (PMC11288421; doi:10.1371/journal.pcbi.1012274)
Supplement: S1 Table — (DOC) [file pcbi.1012274.s001.doc]

***S1 Table. Model comparison and the model selection process for punishment behaviors in pre-test stage in Study 1***

| **Model name** | **Model specification** | **Nested Model** | **Fixed Effects added** |  | **Random Effects** | **Model fit** | | | | **LRT Test against nested** | | |
| --- | --- | --- | --- | --- | --- | --- | --- | --- | --- | --- | --- | --- |
|  |  |  |  |  | **Subjects** | **AIC** | **BIC** | **LL** | **df** | **df** | **X2** | **P value** |
| Model 1 | two-way interactions | - | Culture*Divider+Age+Gender+Education Level + SES |  | (1+Divider \|Subjects) | 3,657.267 | 3,726.194 | -1,817.633 | 11 |  |  |  |
| Model 2 | two-way interactions | Model 1 | Culture*Divider+Age+Gender+Education Level + SES |  | (1+Subjects) | 3,714.366 | 3,770.761 | -1,848.183 | 9 | 2 | 61.099 | 0.000 |
| Model 3 | without two-way interactions | Model 1 | Culture+Divider+Age+Gender+Education Level + SES |  | (1+Divider \|Subjects) | 3,663.788 | 3,726.449 | -1,821.894 | 10 | 1 | 8.521 | 0.004 |

*Note.* This table provides a succession of models that are fit to the data and compared against each other using Likelihood Ratio Tests (LRT). **AIC** – Aikake Information Criterion; **BIC** – Bayesian Information Criterion; **LL** – LogLikelihood; **df** – degrees of freedom; **LRT** – Likeilhood Ratio Test. **X2** – Chi-square. **LRT Test against nested** – results of a Likelihood Ratio Test for the current model against the nested model.
