# Supplementary material for: Reinforcement learning of altruistic punishment differs between cultures and across the lifespan
Source: PLoS Comput Biol. 2024 Jul 11;20(7):e1012274. doi: 10.1371/journal.pcbi.1012274 (PMC11288421; doi:10.1371/journal.pcbi.1012274)
Supplement: S2 Table — (DOC) [file pcbi.1012274.s002.doc]

***S2 Table. Model results for punishment behaviors in pre-test stage in Study 1***

|  | **Estimate** | ***S.E.*** | ***z*** | ***p*** |  |
| --- | --- | --- | --- | --- | --- |
| (Intercept) | –0.665 | (0.175) | –3.794 | < .001 | *** |
| Culture | –0.223 | (0.388) | –0.575 | .565 |  |
| Divider | –1.025 | (0.148) | –6.945 | < .001 | *** |
| Age | –0.030 | (0.020) | –1.500 | .134 |  |
| Gender | 0.183 | (0.355) | 0.517 | .605 |  |
| Education Level | –0.298 | (0.189) | –1.576 | .115 |  |
| SES | 0.123 | (0.077) | 1.609 | .108 |  |
| Culture:Divider | –0.824 | (0.280) | –2.940 | .003 | ** |
| Marginal *R*^2^ | 0.04 | | | | |
| Conditional *R*^2^ | 0.75 | | | | |
| AIC | 3657.27 | | | | |
| BIC | 3726.19 | | | | |
| Num. obs. | 3890 | | | | |
| Num. groups:Subjects | 389 | | | | |
| Var:Subjects (Intercept) | 8.92 | | | | |
| Var:Subjects Divider | 2.40 | | | | |
| Cov:Subjects (Intercept) Divider | –0.89 | | | | |

*Note*. Unstandardized regression coefficients are displayed, with standard errors in parentheses. * *p* < .05. ** *p* < .01. *** *p* < .001.
