# Supplementary material for: Reinforcement learning of altruistic punishment differs between cultures and across the lifespan
Source: PLoS Comput Biol. 2024 Jul 11;20(7):e1012274. doi: 10.1371/journal.pcbi.1012274 (PMC11288421; doi:10.1371/journal.pcbi.1012274)
Supplement: S3 Table — (DOC) [file pcbi.1012274.s003.doc]

S3 Table. Model comparison and the model selection process for punishment behaviors in learning stage in Study 1

| **Model name** | **Model specification** | **Nested Model** | **Fixed Effects added** |  | **Random Effects** | **Model fit** | | | | **LRT Test against nested** | | |
| --- | --- | --- | --- | --- | --- | --- | --- | --- | --- | --- | --- | --- |
|  |  |  |  |  | **Subjects** | **AIC** | **BIC** | **LL** | **df** | **df** | **X2** | **P value** |
| Model 1 | three-way interaction | - | Culture*Divider*Norm+Age+Gender+Educational Level + SES |  | (1+Divider+Index \|Subjects) | convergence warning - item variance close to zero. Removed item intercepts. | | | |  |  |  |
| Model 2 | three-way interaction | - | Culture*Divider*Norm+Age+Gender+Educational Level + SES |  | (1+Divider+Block \|Subjects) | convergence warning - item variance close to zero. Removed item intercepts. | | | |  |  |  |
| Model 3 | three-way interaction | - | Culture*Divider*Norm+Age+Gender+Educational Level + SES |  | (1+Divider \|Subjects) | 18,107.040 | 18,227.909 | -9,038.520 | 15 |  |  |  |
| Model 4 | three-way interaction | Model 3 | Culture*Divider*Norm+Age+Gender+Educational Level + SES |  | (1+Subjects) | 3,714.366 | 3,770.761 | -1,848.183 | 13 | 2 | 1,416.332 | 0.000 |
| **Model 5** | **without three-way interaction** | **Model 3** | **Culture:Divider+Culture:Norm+Divider:Norm+Culture + Divider+Norm+Age+Gender+Educational Level+ SES** |  | **(1+Divider \|Subjects)** | **3,663.788** | **3,726.449** | **-1,821.894** | **14** | **1** | **0.858** | **0.354** |
| Model 6 | without two-way interaction of Culture and Divider | Model 5 | Culture:Norm+Divider:Norm+Culture + Divider+Norm+Age+Gender+Educational Level+ SES |  | (1+Divider |Subjects) | 18,107.678 | 18,212.431 | -9,040.839 | 13 | 1 | 3.781 | 0.052 |

*Note.* This table provides a succession of models that are fit to the data and compared against each other using Likelihood Ratio Tests (LRT). **AIC** – Aikake Information Criterion; **BIC** – Bayesian Information Criterion; **LL** – LogLikelihood; **df** – degrees of freedom; **LRT** – Likeilhood Ratio Test. **X2** – Chi-square. **LRT Test against nested** – results of a Likelihood Ratio Test for the current model against the nested model.
