# Supplementary material for: Reinforcement learning of altruistic punishment differs between cultures and across the lifespan
Source: PLoS Comput Biol. 2024 Jul 11;20(7):e1012274. doi: 10.1371/journal.pcbi.1012274 (PMC11288421; doi:10.1371/journal.pcbi.1012274)
Supplement: S4 Table — (DOC) [file pcbi.1012274.s004.doc]

***S4 Table. Model results for punishment behaviors in learning stage in Study 1***

|  | **Estimate** | ***S.E.*** | ***z*** | ***p*** |  |
| --- | --- | --- | --- | --- | --- |
| (Intercept) | –0.337 | (0.138) | –2.442 | .015 | * |
| Divider | –0.646 | (0.120) | –5.371 | < .001 | *** |
| Culture | 0.499 | (0.306) | 1.629 | .103 |  |
| Norm | –2.511 | (0.277) | –9.067 | < .001 | *** |
| Age | –0.001 | (0.016) | –0.088 | .930 |  |
| Education Level | –0.176 | (0.148) | –1.190 | .234 |  |
| SES | 0.037 | (0.062) | 0.595 | .552 |  |
| Divider:Culture | –0.488 | (0.240) | –2.030 | .042 | * |
| Divider:Norm | 0.144 | (0.238) | 0.604 | .546 |  |
| Culture:Norm | –1.246 | (0.546) | –2.284 | .022 | * |
| Marginal *R*^2^ | 0.15 | | | | |
| Conditional *R*^2^ | 0.75 | | | | |
| AIC | 18104.25 | | | | |
| BIC | 18209.00 | | | | |
| Num. obs. | 23340 | | | | |
| Num. groups:Subjects | 389 | | | | |
| Var:Subjects (Intercept) | 6.89 | | | | |
| Var:Subjects Divider | 4.25 | | | | |
| Cov:Subjects (Intercept) Divider | –0.45 | | | | |

*Note*. Unstandardized regression coefficients are displayed, with standard errors in parentheses. * *p* < .05. ** *p* < .01. *** *p* < .001.
