# Supplementary material for: Reinforcement learning of altruistic punishment differs between cultures and across the lifespan
Source: PLoS Comput Biol. 2024 Jul 11;20(7):e1012274. doi: 10.1371/journal.pcbi.1012274 (PMC11288421; doi:10.1371/journal.pcbi.1012274)
Supplement: S5 Table — (DOC) [file pcbi.1012274.s005.doc]

S5 Table. Study 1 Model Comparison Results

| Model | BICint (Chinese adults and American adults) | BICint (Chinese adults) | BICint (American adults) |
| --- | --- | --- | --- |
| 1α1β | 22392.328 | 11763.1855 | 7442.746659 |
| 2α1β | 19051.666 | 11680.83757 | 7353.983915 |
| 2α2β | 18707.923 | 11378.45105 | 7311.138615 |
| 4α1β | 18126.9108 | 10850.99257 | 7292.342558 |
| 4α2β | 18033.612 | 10748.68646 | 7255.491161 |
| **4α2β + bias** | **17895.3254** | **10652.86653** | **7253.36127** |

*Note*. BICint: integrated Bayesian Information Criterion. In study 1, models with separate α for ingroup and outgroup dividers across two blocks and separate β for dividers (4α2β **+ bias** model) provided the best fit for participants' choices across different samples.
