# Supplementary material for: Reinforcement learning of altruistic punishment differs between cultures and across the lifespan
Source: PLoS Comput Biol. 2024 Jul 11;20(7):e1012274. doi: 10.1371/journal.pcbi.1012274 (PMC11288421; doi:10.1371/journal.pcbi.1012274)
Supplement: S6 Table — (DOC) [file pcbi.1012274.s006.doc]

|  | Simulated | | | | | |
| --- | --- | --- | --- | --- | --- | --- |
| Recovered | αin-1stblock | αout-1stblock | αin-2ndblock | αout-2ndblock | β_in_ | βout |
| αin-1stblock | **0.555** | -0.007 | -0.042 | 0.000 | -0.160 | -0.051 |
| αout-1stblock | -0.005 | **0.411** | 0.003 | -0.035 | 0.012 | -0.103 |
| αin-2ndblock | -0.028 | 0.007 | **0.451** | -0.005 | -0.055 | 0.071 |
| αout-2ndblock | -0.010 | -0.055 | 0.005 | **0.424** | -0.002 | -0.034 |
| β_in_ | -0.062 | 0.005 | -0.043 | 0.010 | **0.755** | 0.134 |
| βout | -0.017 | -0.225 | 0.010 | -0.072 | 0.003 | **0.441** |

S6 Table. Parameter Recovery Results
