# Supplementary material for: Reinforcement learning of altruistic punishment differs between cultures and across the lifespan
Source: PLoS Comput Biol. 2024 Jul 11;20(7):e1012274. doi: 10.1371/journal.pcbi.1012274 (PMC11288421; doi:10.1371/journal.pcbi.1012274)
Supplement: S7 Table — (DOC) [file pcbi.1012274.s007.doc]

**S7 Table. Model comparison and the model selection process for learning rates in Study 1**

| **Model name** | **Model specification** | **Nested Model** | **Fixed Effects added** |  | **Random Effects** | **Model fit** | | | | **LRT Test against nested** | | |
| --- | --- | --- | --- | --- | --- | --- | --- | --- | --- | --- | --- | --- |
|  |  |  |  |  | **Subjects** | **AIC** | **BIC** | **LL** | **df** | **df** | **X2** | **P value** |
| Model 1 | three-way interaction | - | Culture*Divider*Norm+Age+Gender+Educational Level + SES |  | (1+Divider*Block \|Subjects) | convergence warning - item variance close to zero. Removed item intercepts. | | | |  |  |  |
| Model 2 | three-way interaction | Model 1 | Culture*Divider*Norm+Age+Gender+Educational Level + SES |  | (1+Divider+Block \|Subjects) | -554.165 | -452.517 | 296.083 | 19 |  |  |  |
| Model 3 | three-way interaction | Model 2 | Culture*Divider*Norm+Age+Gender+Educational Level + SES |  | (1+Divider \|Subjects) | -519.867 | -434.269 | 275.933 | 16 | 3 | 40.298 | 0.000 |
| Model 4 | three-way interaction | Model 2 | Culture*Divider*Norm+Age+Gender+Educational Level + SES |  | (1+Block \|Subjects) | -550.708 | -465.110 | 291.354 | 16 | 3 | 9.457 | 0.024 |
| **Model 5** | **without three-way interaction** | Model 2 | **Culture:Divider+Culture:Norm+Divider:Norm+Culture + Divider+Norm+Age+Gender+Educational Level+ SES** |  | **(1+Divider+Block \|Subjects)** | **-556.126** | **-459.828** | **296.063** | **18** | **1** | **0.039** | **0.843** |
| Model 6 | without two-way interaction of Culture and Divider | Model 5 | Culture:Norm+Divider:Norm+Culture + Divider+Norm+Age+Gender+Educational Level+ SES |  | (1+Divider+Block |Subjects) | -551.536 | -460.588 | 292.768 | 17 | 1 | 6.590 | 0.010 |

*Note.* This table provides a succession of models that are fit to the data and compared against each other using Likelihood Ratio Tests (LRT). **AIC** – Aikake Information Criterion; **BIC** – Bayesian Information Criterion; **LL** – LogLikelihood; **df** – degrees of freedom; **LRT** – Likeilhood Ratio Test. **X2** – Chi-square. **LRT Test against nested** – results of a Likelihood Ratio Test for the current model against the nested model.
