# Supplementary material for: Reinforcement learning of altruistic punishment differs between cultures and across the lifespan
Source: PLoS Comput Biol. 2024 Jul 11;20(7):e1012274. doi: 10.1371/journal.pcbi.1012274 (PMC11288421; doi:10.1371/journal.pcbi.1012274)
Supplement: S8 Table — (DOC) [file pcbi.1012274.s008.doc]

***S8 Table. Model results for learning rates in Study 1***

|  | **Estimate** | ***S.E.*** | ***df*** | ***t*** | ***p*** |  |
| --- | --- | --- | --- | --- | --- | --- |
| (Intercept) | 0.275 | (0.010) | 381.009 | 27.095 | < .001 | *** |
| Divider | –0.035 | (0.014) | 386.004 | –2.616 | .009 | ** |
| Culture | 0.005 | (0.022) | 381.034 | 0.249 | .803 |  |
| Norm | 0.016 | (0.014) | 381.010 | 1.149 | .251 |  |
| Age | 0.001 | (0.001) | 381.006 | 0.992 | .322 |  |
| Gender | –0.015 | (0.015) | 381.006 | –1.012 | .312 |  |
| SES | –0.000 | (0.003) | 381.006 | –0.036 | .971 |  |
| Education Level | 0.010 | (0.008) | 381.006 | 1.309 | .191 |  |
| Divider:Culture | –0.049 | (0.019) | 386.004 | –2.569 | .011 | * |
| Divider:Norm | –0.040 | (0.019) | 386.004 | –2.119 | .035 | * |
| Culture:Norm | 0.078 | (0.028) | 381.006 | 2.736 | .007 | ** |
| Marginal *R*^2^ | 0.04 | | | | | |
| Conditional *R*^2^ | 0.44 | | | | | |
| AIC | –382.26 | | | | | |
| BIC | –285.96 | | | | | |
| Num. obs. | 1556 | | | | | |
| Num. groups: Subjects | 389 | | | | | |
| Var: Subjects (Intercept) | 0.01 | | | | | |
| Var: Subjects Divider | 0.01 | | | | | |
| Var: Subjects Block | 0.02 | | | | | |
| Cov: Subjects (Intercept) Divider | 0.00 | | | | | |
| Cov: Subjects (Intercept) Block | 0.01 | | | | | |
| Cov: Subjects Divider Block | 0.00 | | | | | |
| Var: Residual | 0.03 | | | | | |

*Note*. Unstandardized regression coefficients are displayed, with standard errors in parentheses.* *p* < .05. ** *p* < .01. *** *p* < .001.
