# Supplementary material for: Reinforcement learning of altruistic punishment differs between cultures and across the lifespan
Source: PLoS Comput Biol. 2024 Jul 11;20(7):e1012274. doi: 10.1371/journal.pcbi.1012274 (PMC11288421; doi:10.1371/journal.pcbi.1012274)
Supplement: S9 Table — (DOC) [file pcbi.1012274.s009.doc]

| **Model name** | **Model specification** | **Nested Model** | **Fixed Effects added** |  | **Random Effects** | **Model fit** | | | | **LRT Test against nested** | | |
| --- | --- | --- | --- | --- | --- | --- | --- | --- | --- | --- | --- | --- |
|  |  |  |  |  | **Subjects** | **AIC** | **BIC** | **LL** | **df** | **df** | **X2** | **P value** |
| Model 1 | three-way interaction | - | Culture*Divider*Norm+Age+Gender+Educational Level + SES |  | (1 \| Subjects) | 886.04 | 946.58 | -430.02 | 13 |  |  |  |
| **Model 2** | **without three-way interaction** | **Model 1** | **Culture:Divider+Culture:Norm+Divider:Norm+Culture + Divider+Norm+Age+Gender+Educational Level+ SES** |  | ( 1\| Subjects) | 886.06 | 941.94 | -431.03 | 12 | 1 | 2.020 | 0.155 |
| Model 3 | without two-way interaction of Culture and Divider | Model 2 | Culture:Norm+Divider:Norm+Culture + Divider+Norm+Age+Gender+Educational Level+ SES |  | (1 \| Subjects) | 890.18 | 941.41 | -434.09 | 11 | 1 | 6.125 | 0.013 |

S9 Table. Model comparison and the model selection process for temperature in Study 1

*Note.* This table provides a succession of models that are fit to the data and compared against each other using Likelihood Ratio Tests (LRT). **AIC** – Aikake Information Criterion; **BIC** – Bayesian Information Criterion; **LL** – LogLikelihood; **df** – degrees of freedom; **LRT** – Likeilhood Ratio Test. **X2** – Chi-square. **LRT Test against nested** – results of a Likelihood Ratio Test for the current model against the nested model.
