# Supplementary material for: Reinforcement learning of altruistic punishment differs between cultures and across the lifespan
Source: PLoS Comput Biol. 2024 Jul 11;20(7):e1012274. doi: 10.1371/journal.pcbi.1012274 (PMC11288421; doi:10.1371/journal.pcbi.1012274)
Supplement: S10 Table — (DOC) [file pcbi.1012274.s010.doc]

**S10 Table. Model results for temperature in Study 1**

|  | **Estimate** | ***S.E.*** | ***df*** | ***t*** | ***p*** |  |
| --- | --- | --- | --- | --- | --- | --- |
| (Intercept) | 0.344 | (0.023) | 381.000 | 15.265 | < .001 | *** |
| Divider | –0.211 | (0.042) | 386.000 | –4.995 | < .001 | *** |
| Norm | –0.001 | (0.032) | 381.000 | –0.026 | .980 |  |
| Culture | 0.138 | (0.048) | 381.000 | 2.878 | .004 | ** |
| Age | –0.002 | (0.002) | 381.000 | –1.038 | .300 |  |
| SES | 0.001 | (0.007) | 381.000 | 0.135 | .892 |  |
| Gender | –0.024 | (0.033) | 381.000 | –0.734 | .463 |  |
| Education Level | 0.028 | (0.017) | 381.000 | 1.683 | .093 | . |
| Divider:Norm | 0.106 | (0.059) | 386.000 | 1.786 | .075 | . |
| Divider:Culture | –0.148 | (0.060) | 386.000 | –2.475 | .014 | * |
| Norm:Culture | –0.056 | (0.063) | 381.000 | –0.886 | .376 |  |
| Marginal *R*^2^ | 0.07 | | | | | |
| Conditional *R*^2^ | 0.12 | | | | | |
| AIC | 950.95 | | | | | |
| BIC | 1011.48 | | | | | |
| Num. obs. | 778 | | | | | |
| Num. groups:Subjects | 389 | | | | | |
| Var:Subjects (Intercept) | 0.01 | | | | | |
| Var: Residual | 0.17 | | | | | |

*Note*. Unstandardized regression coefficients are displayed, with standard errors in parentheses.* *p* < .05. ** *p* < .01. *** *p* < .001.
