# Supplementary material for: Reinforcement learning of altruistic punishment differs between cultures and across the lifespan
Source: PLoS Comput Biol. 2024 Jul 11;20(7):e1012274. doi: 10.1371/journal.pcbi.1012274 (PMC11288421; doi:10.1371/journal.pcbi.1012274)
Supplement: S11 Table — (DOC) [file pcbi.1012274.s011.doc]

***S11 Table. Model comparison and the model selection process for punishment behaviors in pre-test stage in Study 2***

| **Model name** | **Model specification** | **Nested Model** | **Fixed Effects added** |  | **Random Effects** | **Model fit** | | | | **LRT Test against nested** | | |
| --- | --- | --- | --- | --- | --- | --- | --- | --- | --- | --- | --- | --- |
|  |  |  |  |  | **Subjects** | **AIC** | **BIC** | **LL** | **df** | **df** | **X2** | **P value** |
| **Model 1** | **two-way interactions** | **-** | **Age*Divider+Gender+Educational Level + SES** |  | **(1+Divider \|Subjects)** | **4642** | **4699.3** | **-2312.0** | **9** |  |  |  |
| Model 2 | two-way interactions | Model 1 | Age*Divider+Gender+Educational Level + SES |  | (1+Subjects) | 4751 | 4795.6 | 4751 | 7 | 2 | 112.96 | 0.000 |
| Model 3 | without two-way interactions of Age and Divider | Model 1 | Age+Divider+Age+Gender+Educational Level + SES |  | (1+Divider \|Subjects) | 4642.5 | 4693.4 | -2313.2 | 8 | 1 | 8.521 | 0.120 |

*Note.* This table provides a succession of models that are fit to the data and compared against each other using Likelihood Ratio Tests (LRT). **AIC** – Aikake Information Criterion; **BIC** – Bayesian Information Criterion; **LL** – LogLikelihood; **df** – degrees of freedom; **LRT** – Likeilhood Ratio Test. **X2** – Chi-square. **LRT Test against nested** – results of a Likelihood Ratio Test for the current model against the nested model.
