# Supplementary material for: Reinforcement learning of altruistic punishment differs between cultures and across the lifespan
Source: PLoS Comput Biol. 2024 Jul 11;20(7):e1012274. doi: 10.1371/journal.pcbi.1012274 (PMC11288421; doi:10.1371/journal.pcbi.1012274)
Supplement: S12 Table — (DOC) [file pcbi.1012274.s012.doc]

**S12 Table. Model results for punishment behaviors in pre-test stage in Study 2**

|  | **Estimate** | ***S.E.*** | ***z*** | ***p*** |  |
| --- | --- | --- | --- | --- | --- |
| (Intercept) | –0.271 | (0.115) | –2.362 | .018 | * |
| Divider | –1.306 | (0.128) | –10.172 | < .001 | *** |
| Age | –0.017 | (0.014) | –1.274 | .203 |  |
| Gender | 0.682 | (0.228) | 2.992 | .003 | ** |
| SES | 0.109 | (0.053) | 2.066 | .039 | * |
| Divider:Age | –0.024 | (0.015) | –1.556 | .120 |  |
| Marginal *R*^2^ | 0.07 | | | | |
| Conditional *R*^2^ | 0.63 | | | | |
| AIC | 4642.03 | | | | |
| BIC | 4699.33 | | | | |
| Num. obs. | 4300 | | | | |
| Num. groups:Subjects | 430 | | | | |
| Var:Subjects (Intercept) | 4.31 | | | | |
| Var:Subjects Divider | 2.88 | | | | |
| Cov:Subjects (Intercept) Divider | –0.88 | | | | |

*Note*. Unstandardized regression coefficients are displayed, with standard errors in parentheses. * *p* < .05. ** *p* < .01. *** *p* < .001.
