# Supplementary material for: Reinforcement learning of altruistic punishment differs between cultures and across the lifespan
Source: PLoS Comput Biol. 2024 Jul 11;20(7):e1012274. doi: 10.1371/journal.pcbi.1012274 (PMC11288421; doi:10.1371/journal.pcbi.1012274)
Supplement: S13 Table — (DOC) [file pcbi.1012274.s013.doc]

S13 Table. Model comparison and the model selection process for punishment behaviors in learning stage in Study 2

| **Model name** | **Model specification** | **Nested Model** | **Fixed Effects added** |  | **Random Effects** | **Model fit** | | | | **LRT Test against nested** | | |
| --- | --- | --- | --- | --- | --- | --- | --- | --- | --- | --- | --- | --- |
|  |  |  |  |  | **Subjects** | **AIC** | **BIC** | **LL** | **df** | **df** | **X2** | **P value** |
| Model 1 | three-way interaction |  | Age*Divider*Norm+Gender+Educational Level + SES |  | (1+Divider*Block \|Subjects) | convergence warning - item variance close to zero. Removed item intercepts. | | | |  |  |  |
| Model 2 | three-way interaction |  | Age*Divider*Norm+Gender+Educational Level + SES |  | (1+Divider*Index \|Subjects) | convergence warning - item variance close to zero. Removed item intercepts. | | | |  |  |  |
| Model 3 | three-way interaction | - | Age*Divider*Norm+Gender+Educational Level + SES |  | (1+Divider+Index \|Subjects) | 21,043.484 | 21,165.856 | -10,506.742 | 15 |  |  |  |
| Model 4 | three-way interaction | - | Age*Divider*Norm+Gender+Educational Level + SES |  | (1+Divider+Block \|Subjects) | 21,171.899 | 21,294.271 | -10,570.950 | 15 |  |  |  |
| Model 5 | three-way interaction | Model 3 | Age*Divider*Norm+Gender+Educational Level + SES |  | (1+Divider \|Subjects) | 21,260.463 | 21,358.360 | -10,618.231 | 12 | 3.000 | 222.979 | 0.000 |
| **Model 6** | **without three-way interaction** | **Model 3** | **Age:Divider+Age:Norm+Divider:Norm+Divider+Norm+Age+Gender+Educational Level+ SES** |  | (1+Divider+Index \|Subjects) | 21,043.955 | 21,158.169 | -10,507.978 | **14** | **1** | **2.471** | **0.116** |
| Model 7 | without two-way interaction of Age and Divider | Model 6 | Age:Norm+Divider:Norm+ Age+Divider+Norm+Age+Gender+Educational Level+ SES |  | (1+Divider+Index |Subjects) | 21,045.994 | 21,152.049 | -10,509.997 | 13 | 1 | 4.039 | 0.044 |

*Note.* This table provides a succession of models that are fit to the data and compared against each other using Likelihood Ratio Tests (LRT). **AIC** – Aikake Information Criterion; **BIC** – Bayesian Information Criterion; **LL** – LogLikelihood; **df** – degrees of freedom; **LRT** – Likeilhood Ratio Test. **X2** – Chi-square. **LRT Test against nested** – results of a Likelihood Ratio Test for the current model against the nested model.
