# Supplementary material for: Reinforcement learning of altruistic punishment differs between cultures and across the lifespan
Source: PLoS Comput Biol. 2024 Jul 11;20(7):e1012274. doi: 10.1371/journal.pcbi.1012274 (PMC11288421; doi:10.1371/journal.pcbi.1012274)
Supplement: S14 Table — (DOC) [file pcbi.1012274.s014.doc]

**S14 Table. Model results for punishment behaviors in learning stage in Study 2**

|  | **Estimate** | ***S.E.*** | ***z*** | ***p*** |  |
| --- | --- | --- | --- | --- | --- |
| (Intercept) | –0.082 | (0.086) | –0.959 | .338 |  |
| Norm | –3.070 | (0.197) | –15.618 | < .001 | *** |
| Age | –0.012 | (0.010) | –1.103 | .270 |  |
| Divider | –0.967 | (0.122) | –7.926 | < .001 | *** |
| SES | –0.015 | (0.040) | –0.373 | .709 |  |
| Norm:Age | 0.059 | (0.021) | 2.829 | .005 | ** |
| Norm:Divider | 0.069 | (0.266) | 0.259 | .795 |  |
| Age:Divider | 0.029 | (0.014) | 2.042 | .041 | * |
| Marginal *R*^2^ | 0.25 | | | | |
| Conditional *R*^2^ | 0.69 | | | | |
| AIC | 21043.96 | | | | |
| BIC | 21158.17 | | | | |
| Num. obs. | 25800 | | | | |
| Num. groups:Subjects | 430 | | | | |
| Var:Subjects (Intercept) | 2.83 | | | | |
| Var:Subjects Divider | 5.36 | | | | |
| Var:Subjects Index | 0.00 | | | | |
| Cov:Subjects (Intercept) Divider | –0.51 | | | | |
| Cov:Subjects (Intercept) Index | –0.02 | | | | |
| Cov:Subjects Divider Index | 0.00 | | | | |

*Note*. Unstandardized regression coefficients are displayed, with standard errors in parentheses.* *p* < .05. ** *p* < .01. *** *p* < .001.
