# Supplementary material for: Reinforcement learning of altruistic punishment differs between cultures and across the lifespan
Source: PLoS Comput Biol. 2024 Jul 11;20(7):e1012274. doi: 10.1371/journal.pcbi.1012274 (PMC11288421; doi:10.1371/journal.pcbi.1012274)
Supplement: S15 Table — (DOC) [file pcbi.1012274.s015.doc]

S15 Table. Study 2 Model Comparison Results

| Model | BICint (Chinese adults and adolescents) | BICint (Chinese adolescents) | BICint (Chinese adults) |
| --- | --- | --- | --- |
| 1α1β | 26245.93105 | 16319.882 | 11763.1855 |
| 2α1β | 25998.20984 | 15739.299 | 11680.83757 |
| 2α2β | 25243.45236 | 15292.34 | 11378.45105 |
| 4α1β | 24780.44789 | 14761.42903 | 10850.99257 |
| 4α2β | 24315.47203 | 14578.871 | 10748.68646 |
| **4α2β + bias** | **24022.27025** | **13326.38455** | **10652.86653** |

Note. BICint: integrated Bayesian Information Criterion. In study 2, models with separate α for ingroup and outgroup dividers across two blocks and separate β for dividers (4α2β **+ bias** model) provided the best fit for participants' choices across different samples
