# Supplementary material for: Reinforcement learning of altruistic punishment differs between cultures and across the lifespan
Source: PLoS Comput Biol. 2024 Jul 11;20(7):e1012274. doi: 10.1371/journal.pcbi.1012274 (PMC11288421; doi:10.1371/journal.pcbi.1012274)
Supplement: S16 Table — (DOC) [file pcbi.1012274.s016.doc]

**S16 Table. Model comparison and the model selection process for learning rates in Study 2**

| **Model name** | **Model specification** | **Nested Model** | **Fixed Effects added** |  | **Random Effects** | **Model fit** | | | | **LRT Test against nested** | | |
| --- | --- | --- | --- | --- | --- | --- | --- | --- | --- | --- | --- | --- |
|  |  |  |  |  | **Subjects** | **AIC** | **BIC** | **LL** | **df** | **df** | **X2** | **P value** |
| Model 1 | three-way interaction | - | Age*Divider*Norm+Gender+ SES |  | (1+Divider*Block \|Subjects) | convergence warning - item variance close to zero. Removed item intercepts. | | | |  |  |  |
| Model 2 | three-way interaction | Model 1 | Age*Divider*Norm+Gender+ SES |  | (1+Divider+Block \|Subjects) | -589.469 | -496.817 | 311.734 | 17 |  |  |  |
| Model 3 | three-way interaction | Model 2 | Age*Divider*Norm+Gender+ SES |  | (1+Divider \|Subjects) | --568.944 | -492.643 | 298.472 | 14 | 3 | 26.525 | 0.000 |
| Model 4 | three-way interaction | Model 2 | Age*Divider*Norm+Gender+ SES |  | (1+Block \|Subjects) | --593.686 | -517.385 | 310.843 | 14 | 3 | 1.783 | 0.619 |
| **Model 5** | **without three-way interaction** | Model 4 | **Age:Divider+Culture:Norm+Divider:Norm+Age+ Divider+Norm+Gender+Educational Level+ SES** |  | (1+Block \|Subjects) **)** | --593.833 | -522.982 | 309.916 | **13** | **1** | **1.853** | **0.173** |
| Model 6 | without two-way interaction of Age and Divider | Model 5 | Age:Norm+Divider:Norm+Age+ Divider+Norm+Gender+Educational Level+ SES |  | (1+Block |Subjects) **)** | --586.879 | -521.478 | 305.439 | 12 | 1 | 8.954 | 0.003 |

*Note.* This table provides a succession of models that are fit to the data and compared against each other using Likelihood Ratio Tests (LRT). **AIC** – Aikake Information Criterion; **BIC** – Bayesian Information Criterion; **LL** – LogLikelihood; **df** – degrees of freedom; **LRT** – Likeilhood Ratio Test. **X2** – Chi-square. **LRT Test against nested** – results of a Likelihood Ratio Test for the current model against the nested model.
