# Supplementary material for: Reinforcement learning of altruistic punishment differs between cultures and across the lifespan
Source: PLoS Comput Biol. 2024 Jul 11;20(7):e1012274. doi: 10.1371/journal.pcbi.1012274 (PMC11288421; doi:10.1371/journal.pcbi.1012274)
Supplement: S17 Table — (DOC) [file pcbi.1012274.s017.doc]

S17 Table. Model results for learning rates in Study 2

|  | **Estimate** | **S.E.** | **df** | **t** | **p** |  |
| --- | --- | --- | --- | --- | --- | --- |
| (Intercept) | –0.032 | (0.036) | 427.734 | –0.876 | .381 |  |
| Divider | –0.310 | (0.054) | 857.003 | –5.717 | < .001 | *** |
| Age_s | –0.125 | (0.033) | 423.991 | –3.824 | < .001 | *** |
| Gender | –0.032 | (0.053) | 423.991 | –0.606 | .545 |  |
| Norm | 0.064 | (0.051) | 423.991 | 1.243 | .215 |  |
| Block | 0.687 | (0.046) | 429.000 | 14.907 | < .001 | *** |
| SES | 0.017 | (0.012) | 423.991 | 1.429 | .154 |  |
| Divider:Age_s | –0.116 | (0.039) | 857.003 | –2.995 | .003 | ** |
| Divider:Norm | –0.049 | (0.078) | 857.003 | –0.631 | .528 |  |
| Age_s:Norm | 0.084 | (0.053) | 423.991 | 1.578 | .115 |  |
| Marginal *R*^2^ | 0.16 | | | | | |
| Conditional *R*^2^ | 0.35 | | | | | |
| AIC | 4586.67 | | | | | |
| BIC | 4662.98 | | | | | |
| Num. obs. | 1720 | | | | | |
| Num. groups:Subjects | 430 | | | | | |
| Var:Subjects (Intercept) | 0.13 | | | | | |
| Var:Subjects Block | 0.26 | | | | | |
| Cov:Subjects (Intercept) Block | 0.07 | | | | | |
| Var: Residual | 0.65 | | | | | |

*Note*. Unstandardized regression coefficients are displayed, with standard errors in parentheses. * *p* < .05. ** *p* < .01. *** *p* < .001.
