# Supplementary material for: Reinforcement learning of altruistic punishment differs between cultures and across the lifespan
Source: PLoS Comput Biol. 2024 Jul 11;20(7):e1012274. doi: 10.1371/journal.pcbi.1012274 (PMC11288421; doi:10.1371/journal.pcbi.1012274)
Supplement: S18 Table — (DOC) [file pcbi.1012274.s018.doc]

**S18 Table. Model comparison and the model selection process for adolescents’ learning rates in Study 2**

| **Model name** | **Model specification** | **Nested Model** | **Fixed Effects added** |  | **Random Effects** | **Model fit** | | | | **LRT Test against nested** | | |
| --- | --- | --- | --- | --- | --- | --- | --- | --- | --- | --- | --- | --- |
|  |  |  |  |  | **Subjects** | **AIC** | **BIC** | **LL** | **df** | **df** | **X2** | **P value** |
| Model 1 | three-way interaction | - | Age*Divider*Norm+Gender+ SES |  | (1+Divider*Block \|Subjects) | convergence warning - item variance close to zero. Removed item intercepts. | | | |  |  |  |
| Model 2 | three-way interaction | Model 1 | Age*Divider*Norm+Gender+ SES |  | (1+Divider+Block \|Subjects) | -426.405 | -333.753 | 230.202 | 17 |  |  |  |
| Model 3 | three-way interaction | Model 2 | Age*Divider*Norm+Gender+ SES |  | (1+Divider \|Subjects) | boundary (singular) fit | | | |  |  |  |
| Model 4 | three-way interaction | Model 2 | Age*Divider*Norm+Gender+ SES |  | (1+Block \|Subjects) | -430.243 | -353.942 | 229.122 | 14 | 3 | 2.162 | 0.540 |
| **Model 5** | **without three-way interaction** | **Model 4** | **Age:Divider+Culture:Norm+Divider:Norm+Age+ Divider+Norm+Gender+Educational Level+ SES** |  | **(1+Block \|Subjects) )** | **-431.171** | **-360.320** | **228.586** | **13** | **4** | **3.234** | **0.520** |
| Model 6 | without two-way interaction of Age and Divider | Model 5 | Age:Norm+Divider:Norm+Age+ Divider+Norm+Gender+Educational Level+ SES |  | (1+Block |Subjects) ) | -419.932 | -354.531 | 221.966 | 12 | 1 | 13.240 | 0.000 |

*Note.* This table provides a succession of models that are fit to the data and compared against each other using Likelihood Ratio Tests (LRT). **AIC** – Aikake Information Criterion; **BIC** – Bayesian Information Criterion; **LL** – LogLikelihood; **df** – degrees of freedom; **LRT** – Likeilhood Ratio Test. **X2** – Chi-square. **LRT Test against nested** – results of a Likelihood Ratio Test for the current model against the nested model.
