# Supplementary material for: Reinforcement learning of altruistic punishment differs between cultures and across the lifespan
Source: PLoS Comput Biol. 2024 Jul 11;20(7):e1012274. doi: 10.1371/journal.pcbi.1012274 (PMC11288421; doi:10.1371/journal.pcbi.1012274)
Supplement: S19 Table — (DOC) [file pcbi.1012274.s019.doc]

***S19 Table. Model results for adolescents’ learning rates in Study 2***

|  | **Estimate** | ***S.E.*** | ***df*** | ***t*** | ***p*** |  |
| --- | --- | --- | --- | --- | --- | --- |
| (Intercept) | 0.423 | (0.010) | 912.992 | 40.918 | < .001 | *** |
| Gender | –0.011 | (0.012) | 424.002 | –0.908 | .364 |  |
| SES | 0.002 | (0.003) | 424.002 | 0.786 | .432 |  |
| Divideringroup:Norm | –0.094 | (0.014) | 913.972 | –6.518 | < .001 | *** |
| Divideroutgroup:Norm | –0.023 | (0.014) | 913.972 | –1.600 | .110 |  |
| Divideringroup:Norm | –0.079 | (0.013) | 858.013 | –6.312 | < .001 | *** |
| Norm:Age group | 0.086 | (0.016) | 424.002 | 5.370 | < .001 | *** |
| Norm:Age group | 0.022 | (0.017) | 424.002 | 1.293 | .197 |  |
| Marginal *R*^2^ | 0.05 | | | | | |
| Conditional *R*^2^ | 0.35 | | | | | |
| AIC | –360.54 | | | | | |
| BIC | –295.14 | | | | | |
| Num. obs. | 1720 | | | | | |
| Num. groups:Subjects | 430 | | | | | |
| Var:Subjects (Intercept) | 0.01 | | | | | |
| Var:Subjects Block | 0.04 | | | | | |
| Cov:Subjects (Intercept) Block | 0.00 | | | | | |
| Var: Residual | 0.03 | | | | | |

*Note*. Unstandardized regression coefficients are displayed, with standard errors in parentheses. * *p* < .05. ** *p* < .01. *** *p* < .001.
