# Supplementary material for: Reinforcement learning of altruistic punishment differs between cultures and across the lifespan
Source: PLoS Comput Biol. 2024 Jul 11;20(7):e1012274. doi: 10.1371/journal.pcbi.1012274 (PMC11288421; doi:10.1371/journal.pcbi.1012274)
Supplement: S20 Table — (DOC) [file pcbi.1012274.s020.doc]

**S20 Table. Model comparison and the model selection process for temperature in Study 2**

| **Model name** | **Model specification** | **Nested Model** | **Fixed Effects added** |  | **Random Effects** | **Model fit** | | | | | | **LRT Test against nested** | | |
| --- | --- | --- | --- | --- | --- | --- | --- | --- | --- | --- | --- | --- | --- | --- |
|  |  |  |  |  | **Subjects** | **AIC** | **BIC** | | **LL** | | **df** | **df** | **X2** | **P value** |
| Model 1 | three-way interaction | - | Age*Divider*Norm+Gender+ SES |  | (1 \| Subjects) | boundary (singular) fit | | | | | |  |  |  |
| Model 2 | without three-way interaction | - | Age:Divider+ Age:Norm+Divider:Norm+Age + Divider+Norm+Gender+Educational Level+ SES |  | ( 1\| Subjects) | boundary (singular) fit | | | | | |  |  |  |
| **Model 3** | **without two-way interaction** | **Model 1** | **Age + Divider+Norm+Gender+Educational Level+ SES** |  | ( 1| Subjects) | 2172.696 | | 2201.238 | **-1069.1** | **6** | |  |  |  |

*Note.* This table provides a succession of models that are fit to the data and compared against each other using Likelihood Ratio Tests (LRT). **AIC** – Aikake Information Criterion; **BIC** – Bayesian Information Criterion; **LL** – LogLikelihood; **df** – degrees of freedom; **LRT** – Likeilhood Ratio Test. **X2** – Chi-square. **LRT Test against nested** – results of a Likelihood Ratio Test for the current model against the nested model.
