# Supplementary material for: Reinforcement learning of altruistic punishment differs between cultures and across the lifespan
Source: PLoS Comput Biol. 2024 Jul 11;20(7):e1012274. doi: 10.1371/journal.pcbi.1012274 (PMC11288421; doi:10.1371/journal.pcbi.1012274)
Supplement: S21 Table — (DOC) [file pcbi.1012274.s021.doc]

**S21 Table. Model results for temperature in Study 2**

|  | **Estimate** | ***S.E.*** | ***df*** | ***t*** | ***p*** |  |
| --- | --- | --- | --- | --- | --- | --- |
| (Intercept) | 0.781 | (0.084) | 856.000 | 9.246 | < .001 | *** |
| Divider | –0.216 | (0.057) | 856.000 | –3.766 | < .001 | *** |
| Norm | –0.088 | (0.057) | 856.000 | –1.533 | .126 |  |
| Age | –0.009 | (0.003) | 856.000 | –2.776 | .006 | ** |
| Marginal *R*^2^ | 0.03 | | | | | |
| Conditional *R*^2^ | 0.03 | | | | | |
| AIC | 2172.70 | | | | | |
| BIC | 2201.24 | | | | | |
| Num. obs. | 860 | | | | | |
| Num. groups:Subjects | 430 | | | | | |
| Var:Subjects (Intercept) | 0.00 | | | | | |
| Var: Residual | 0.71 | | | | | |

*Note*. Unstandardized regression coefficients are displayed, with standard errors in parentheses. * *p* < .05. ** *p* < .01. *** *p* < .001.
